# Phylogenies of the 16S rRNA gene and its hypervariable regions lack concordance with core genome phylogenies

**DOI:** 10.1186/s40168-022-01295-y

**Published:** 2022-07-08

**Authors:** Hayley B. Hassler, Brett Probert, Carson Moore, Elizabeth Lawson, Richard W. Jackson, Brook T. Russell, Vincent P. Richards

**Affiliations:** 1 Department of Biological Sciences, College of Science, Clemson University, Clemson, SC 29634 USA; 2 Software Engineer, ITW Hartness, Greenville, SC 29605 USA; 3 School of Mathematical and Statistical Sciences, Clemson University, Clemson, SC 29634 USA

**Keywords:** 16S rRNA gene, Comparative phylogenomics, Microbiome, Diversity metrics, Entropy masking, Recombination, Horizontal gene transfer, Ribosome, Species phylogeny

## Abstract

**Background:**

The 16S rRNA gene is used extensively in bacterial phylogenetics, in species delineation, and now widely in microbiome studies. However, the gene suffers from intragenomic heterogeneity, and reports of recombination and an unreliable phylogenetic signal are accumulating. Here, we compare core gene phylogenies to phylogenies constructed using core gene concatenations to estimate the strength of signal for the 16S rRNA gene, its hypervariable regions, and all core genes at the intra- and inter-genus levels. Specifically, we perform four intra-genus analyses (*Clostridium*, *n* = 65; *Legionella*, *n* = 47; *Staphylococcus*, *n* = 36; and *Campylobacter*, *n* = 17) and one inter-genus analysis [41 core genera of the human gut microbiome (31 families, 17 orders, and 12 classes), *n* = 82].

**Results:**

At both taxonomic levels, the 16S rRNA gene was recombinant and subject to horizontal gene transfer. At the intra-genus level, the gene showed one of the lowest levels of concordance with the core genome phylogeny (50.7% average). Concordance for hypervariable regions was lower still, with entropy masking providing little to no benefit. A major factor influencing concordance was SNP count, which showed a positive logarithmic association. Using this relationship, we determined that 690 ± 110 SNPs were required for 80% concordance (average 16S rRNA gene SNP count was 254). We also found a wide range in 16S-23S-5S rRNA operon copy number among genomes (1–27). At the inter-genus level, concordance for the whole 16S rRNA gene was markedly higher (73.8% — 10th out of 49 loci); however, the most concordant hypervariable regions (V4, V3-V4, and V1-V2) ranked in the third quartile (62.5 to 60.0%).

**Conclusions:**

Ramifications of a poor phylogenetic performance for the 16S rRNA gene are far reaching. For example, in addition to incorrect species/strain delineation and phylogenetic inference, it has the potential to confound community diversity metrics if phylogenetic information is incorporated — for example, with popular approaches such as Faith’s phylogenetic diversity and UniFrac. Our results highlight the problematic nature of these approaches and their use (along with entropy masking) is discouraged. Lastly, the wide range in 16S rRNA gene copy number among genomes also has a strong potential to confound diversity metrics.

Video Abstract

**Supplementary Information:**

The online version contains supplementary material available at 10.1186/s40168-022-01295-y.

## Background

Extensive use of the 16S rRNA gene in phylogenetics was first pioneered by Carl Woese in 1977 to delineate the previously undescribed taxonomic lineage — Archaea [1]. Woese justified the use of the 16S rRNA gene and other rRNA genes (5S and 23S) by highlighting their universality in bacteria and their molecular clock-like nature [2]. An important characteristic that favors the use of the 16S rRNA gene in particular is the presence of multiple conserved/hypervariable regions that allow multiple options for PCR primer design [3]. More recently, the hypervariable regions have gained widespread use in microbiome studies, as in addition to their universality in bacteria, their length in nucleotides is well suited to next-generation sequencing platforms. However, the question of which hypervariable region and/or combination of regions provides optimal results is debated [4–6].

Variations in the nucleotide sequence of the 16S rRNA gene were historically assumed to be more likely a product of speciation and vertical inheritance than horizontal gene transfer (HGT) and/or events of recombination [7–11]. Despite this assumption, accumulating reports provide evidence suggesting that the gene is subject to both these phenomena [12–15]. Several studies have also investigated the intragenomic heterogeneity of multiple copies of the 16S rRNA gene [12, 16, 17]. The prevalence of multiple copies of the gene in a single genome may facilitate PCR-induced chimeras to form between the copies, leading to inaccurate characterizations of bacterial species [18]. Additionally, multiple copies within a genome have the potential to inflate taxonomic abundance and confound measures of microbiome diversity [19, 20].

Alternative phylogenetic approaches to using the 16S rRNA gene for novel species delineation include producing other rRNA gene phylogenies (5S or 23S), combined rRNA gene phylogenies (16S-23S), one or more protein coding gene phylogenies, or a core genome phylogeny (one produced using genes shared among all OTUs). Reports of discordance between phylogenies produced using these approaches and 16S rRNA gene phylogenies are numerous [21–38] and call into question the reliability or strength of the phylogenetic signal for the gene. The ramifications of this unresolved question are far reaching given the extensive use of the gene in many areas of research. For example, in addition to incorrectly delineating new species and phylogenetic position, there are ramifications for microbiome studies. Specifically, popular approaches used to calculate alpha and beta diversity within and among microbial communities such as Faith’s phylogenetic diversity and UniFrac incorporate phylogenetic information [39–41] and these approaches are incorporated into the two most popular microbiome analyses pipelines: Mothur and QIIME2 [42, 43]. Clearly, there is a need to critically evaluate the strength of the phylogenetic signal for the 16S rRNA gene. Here, we take a novel phylogenomic approach that measures concordance between a gene phylogeny and a putative species phylogeny built using genes shared among all taxa (the core genome) to evaluate the level of concordance for the 16S rRNA gene, other rRNA genes, and all single-copy core genes at the intra-genus level in four highly divergent genera (two Gram-positive and two Gram-negative) that contain important pathogens: *Staphylococcus*, *Clostridium*, *Campylobacter*, and *Legionella*. We find that (i) all four genera exhibited evidence for 16S rRNA gene recombination/HGT, (ii) the 16S rRNA gene displayed one of the lowest levels of concordance with the species phylogeny of any gene tested, (iii) hypervariable regions of the 16S rRNA gene showed a decrease in concordance compared to the full gene, (iv) entropy masking provided little to no benefit, (v) protein coding ribosomal genes also displayed low concordance on average, (vi) concordance for any given gene was strongly predicted by alignment single nucleotide polymorphism (SNP) count, and (vii) SNPs from non-ribosomal protein coding genes displayed the strongest concordance while SNPs from rRNA genes showed the weakest concordance. Given the broad taxonomic scope of microbiome studies, we extended our approach to evaluate phylogenetic performance at the inter-genus level. Here, core genes were evaluated using a phylogeny representing 41 core genera of the human gut microbiome. At this evolutionary scale, concordance for the full 16S rRNA gene was improved, ranking in the first quartile with 73.8% concordance (10th out of 49 loci). Although concordance for some hypervariable regions was improved, even the most concordant regions (V4, V3-V4, and V1-V2) ranked in the third quartile with 62.5 to 60.0% concordance.

## Results

### Intra-genus homologous gene clustering and recombination/HGT

For the four intra-genus analyses, we chose four highly divergent and clinically relevant genera as a representation of the range of diversity existing among bacteria. For each genus, we downloaded all available assembled genome sequences and their assembly statistics from the RefSeq genome database at NCBI. Assessing all assemblies, we selected a representative strain for each species within each genus: *Clostridium* (65),* Legionella* (47),* Staphylococcus* (36), and *Campylobacter* (17). Strain selection preference was given to closed genomes and those assemblies with fewer contigs. Strain information and accession numbers regarding the 165 genomes used in our analyses are presented in Table S1.

The first step in our approach required the use of homologous gene clustering to delineate core genes. After paralogs and genes judged subject to recombination and/or HGT were removed (see below), core gene phylogenies were constructed using concatenations of core gene alignments (the species phylogeny, Fig. S1A-H). Phylogenies for each core gene were then separately compared to the species phylogeny and the proportion of bipartition concordance between the two was calculated. Reliability of each species phylogeny was assessed using bootstrapping and all phylogenies showed strong support (Figs. S1A, C, E, and G). We further assessed the reliability of the species phylogenies by comparison to a second core gene phylogeny that represented a consensus of the topologies of each single-copy core gene phylogeny. Each consensus phylogeny was highly concordant with its respective species phylogeny (Figs. S1B, D, F, and H). Specifically, for *Staphylococcus*, the phylogenies were identical; for *Legionella*, the phylogenies differed by two bipartitions (95.6% concordance); for *Clostridium*, the phylogenies differed by four bipartitions (93.5% concordance); and for *Campylobacter*, the phylogenies differed by one bipartition (93.3% concordance). These differences involved minor rearrangements among closely placed taxa.

The homologous gene clustering delineated 120 single-copy core genes for *Clostridium*, 392 for *Legionella*, 604 for *Staphylococcus*, and 495 for *Campylobacter.* We utilized two separate approaches to test for recombination [pairwise homoplasy index (PHI) and single breakpoint (SBP)] and one for HGT (HGTector) [44–46]. The number of genes that exhibited evidence of recombination/HGT for any test was as follows: *Clostridium* = 53 (44.2%), *Legionella* = 51 (13.0%), *Staphylococcus* = 246 (40.7%), and *Campylobacter* = 299 (60.4%) (see Table S2 for a breakdown of each test). After excluding these genes, the following number remained: *Clostridium* = 67 (55.8%), *Legionella* = 341 (87.0%), *Staphylococcus* = 358 (59.3%), and *Campylobacter* = 196 (39.6%).

The 16S rRNA gene exhibited evidence of recombination for the PHI approach in *Campylobacter*, *Legionella*, and *Clostridium*, for the SBP approach in *Campylobacter* and *Legionella*, and negative for both approaches for *Staphylococcus* (Table 1). When evaluating possible HGT events, HGTector can only utilize amino acid sequences. Therefore, given the non-protein coding nature of the 16S rRNA gene, we applied an alternative phylogenetic approach. First, we produced 16S rRNA gene phylogenies including all copies of the gene within all genomes for each genus (the 16S-23S-5S operon can exist as multiple copies within a genome — see Table S3 for frequency distribution among genomes). Then, any gene copy that was monophyletic within a species was considered to be vertically inherited. Alternatively, if a gene copy for a species clustered within a grouping from a second species, HGT for that copy was inferred. This analysis suggested exchange among strains within *Staphylococcus* and *Clostridium* but not *Legionella* or *Campylobacter* (Table S3, Fig. S2A-D). In *Staphylococcus*, three instances of putative HGT were identified (Fig. S2D): the six copies of the 16S rRNA gene in *Staphylococcus pseudintermedius* were not monophyletic, but instead the single copies of *Staphylococcus delphini* and *Staphylococcus intermedius* fell within the grouping. Additionally, the four copies of the gene in *Staphylococcus aureus* fell sporadically within the clade containing the five copies of the gene in *Staphylococcus argenteus*. Finally, one of the six *Staphylococcus condimenti* copies grouped polyphyletically with the five copies of *Staphylococcus carnosus*, suggesting HGT among these species*.* In *Clostridium*, two instances of putative HGT were identified (Fig. S2B): the eight copies of 16S rRNA gene in *Clostridium botulinum* fell sporadically within the clade containing the nine copies of the gene in *Clostridium sporogenes.* The sole copy of the 16S rRNA gene in *Clostridium coskatii* and the nine copies of the gene in *Clostridium ljungdahlii* fell sporadically within the clade containing the nine copies of the gene in *Clostridium autoethanogenum*.

**Table 1:** Recombination and horizontal gene transfer (HGT) test results

	***Campylobacter*** 17 species, 495 core genes, 445 NR, 50 CR			***Clostridium*** 65 species, 120 core genes, 108 NR, 12 CR			***Legionella*** 47 species, 392 core genes, 348 NR, 44, CR			***Staphylococcus*** 36 species, 604 core genes, 556 NR, 48 CR		
Locus	**PHI**	**SBP**	**HGT**	**PHI**	**SBP**	**HGT**	**PHI**	**SBP**	**HGT**	**PHI**	**SBP**	**HGT**
*rpo*A	X	X	X	X	X	X	X	X	X	X	X	✓
*rpo*B	✓	✓	X	✓	X	X	X	X	X	X	X	X
rpoB*	X	X		X	X		X	X		X	X	
*rpo*C	✓	X	X	X	X	✓	X	X	X	✓	X	X
NR (%)	0.38	0.15	0.32	0.11	0.03	0.41	0.01	0.02	0.08	0.09	0.07	0.22
CR (%)	0.12	0.08	0.16	0.08	0.00	0.08	0.05	0.02	0.23	0.02	0.00	0.79
16S	✓	✓	X	✓	X	✓	✓	✓	X	X	X	✓
23S	X	X	X	X	X	✓	X	✓	X	X	X	✓
5S		X	✓		X	✓		X	X		X	✓
16S M	✓	✓		X	X		✓	✓		X	X	
V1-2	✓	X			X		X	X		X	X	
V3		X			X			X			X	
V3 M		X		X	X			X			X	
V3-V4	X	X		X	X		X	X		X	X	
V3-V4 M		X			X		X	X		X	X	
V4	X	X			X		X	X		X	X	
V4 M		X		X	X			X			X	
V5		X			X			X			X	
V6		X			X			X			X	
V7		X			X			X			X	
V8		X			X			X			X	
V9		X			X		NP	NP	NP		X	

*NR* Non-ribosomal genes, *CR* Coding ribosomal genes, *PHI* Pairwise homoplasy index recombination analysis, *SBP* Single break-point recombination analysis, *HGT* HGTector analysis for protein coding genes and our phylogenetic approach to detect HGT for rRNA genes (see text), rpoB* partial *rpo*B sequence based on primers published by Ogier et al. [47], *M* masked locus, *V* hypervariable regions of the 16S rRNA gene, ✓ indicates loci positive for recombination or HGT, X indicates loci negative for recombination or HGT, blank cells indicate where metrics could not be obtained due to the nature of the locus (short gene length for PHI, not protein coding for HGTector, single-copy gene for phylogenetic HGT), NR and CR loci presented as proportion of genes positive for recombination or HGT, *NP* not possible (missing locus)

The 23S rRNA gene tested negative for recombination for both PHI and SBP approaches for all genera with the exception of the PHI approach in *Legionella* (Table 1). For *Clostridium*, 23 genes could not be tested as their gene sequence was missing from the genome — likely the result of rRNA operon truncation (Table S3). For the 5S rRNA gene, there was an insufficient number of informative nucleotide sites within each of the alignments for PHI to run using the default settings. The 5S rRNA gene alignments contained among the fewest number of SNPs when compared to all other genes (Tables S4A-D and S5) and reducing the sliding window used to calculate the PHI statistic from the default of 100 nucleotides to 50 nucleotides enabled the program to run. However, recombination was not detected for any alignment. The gene also tested negative for recombination for the SBP approach in all four genera (Table 1). The analysis for *Campylobacter* was missing three species, again due to missing gene sequence (Table S3). We utilized the same phylogenetic approach as with the 16S rRNA gene to assess putative HGT for both 23S and 5S rRNA genes. These analyses suggested HGT among species in all genera with the exception of *Legionella* for both genes and *Campylobacter* for the 23S rRNA gene (Table 1 and Fig. S2E-L). However, we acknowledge that our phylogenetic approach to detect HGT may be susceptible to error when the underlying phylogenetic signal was weak. This was particularly the case for the 5S rRNA gene, which due to its short length and low SNP count showed very low concordance with the species phylogeny. There was a wide distribution of 16S-23S-5S rRNA operon copy number among genomes for each genus (Fig. S3 and Table S3). For example, *Clostridium* ranged from two to 27 copies. For the other genera, the numbers were as follows: *Campylobacter* (1 to 3), *Legionella* (1 to 4), and *Staphylococcus* (1 to 9). These numbers should be treated as estimates, as the majority of genomes were whole genome shotgun and the operon was frequently truncated at the end of a contig.

### Intra-genus phylogenetic concordance and nucleotide substitution

**Fig. 1:**
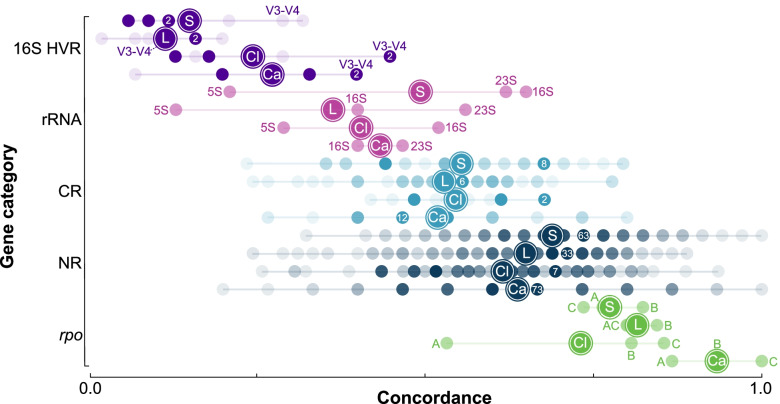
Levels of concordance with the species phylogeny for core genes sorted from lowest (left) to highest (right) for the 16S rRNA gene hypervariable regions (16S HVR, purple), rRNA genes (rRNA, pink), coding ribosomal genes (CR, light blue), non-ribosomal genes (NR, dark blue), and *rpo* genes (*rpo*, green). Colored dots correspond to levels of concordance with the species phylogeny for genes (and hypervariable regions). Degree of shading represents the number of loci observed for each level of concordance. For 16S HVR, SC, and NR, a single dot for each genus contains a number that shows the maximum number of loci observed across all levels of concordance for that genus. This dot will have the darkest level of shading. In all cases, there are multiple dots with this maximum level of shading and maximum loci count. For rRNA and *rpo*, there are only three loci (5S, 16S, 23S and *rpo*A, *rpo*B, *rpo*C) each with a frequency of one and the same degree of shading. The following designations indicate average concordance for each genus: *Staphylococcus* (S), *Legionella* (L), *Clostridium* (Cl), and *Campylobacter* (Ca). Hypervariable regions frequently used in microbiome research (V3-V4) are highlighted. Individual rRNA and *rpo* genes are designated as follows: 16S rRNA gene (16S), 23S rRNA gene (23S), 5S rRNA gene (5S), *rpo*A gene (A), *rpo*B gene (B), and *rpo*C gene (C)

Figure 1 and Tables S4A-D show the distribution of levels of concordance for each gene delineated into five gene categories: non-ribosomal (NR), protein coding ribosomal (CR), rRNA, 16S HVR (16S rRNA gene hypervariable regions — discussed in more detail below), and *rpo* (RNA polymerase genes — discussed in more detail below). Overall, hypervariable regions and rRNA genes had among the lowest concordance. Specifically, concordance for the 16S rRNA gene was 64.7% (*Staphylococcus*), 51.6% (*Clostridium*), 40.0% (*Legionella*), and 46.7% (*Campylobacter*). To place these levels in perspective, for each genus, the following proportion of genes had higher concordance than the 16S rRNA gene: *Staphylococcus* = 59.4%, *Clostridium* = 72.5%, *Legionella* = 96.2%, and *Campylobacter* = 76.2%. The average concordance for protein coding ribosomal genes was intermediate between rRNA genes and non-ribosomal genes (rRNA = 42.3%, CR = 52.6%, NR = 66.0%, Table S5).

To complement our gene ranking approach based on concordance with the species phylogeny, we additionally compared ranking based on gene phylogeny log-likelihood values and ran the approximately unbiased (AU) topology test [48]. Results showed the likelihood values to be highly concordant with concordance levels (Table S4A-D). For *Campylobacter*, four phylogenies showed no significant difference from the species phylogeny and these phylogenies showed the highest concordance (100%) (Table S4A-D). For *Clostridium*, one phylogeny showed no significant difference and showed the highest concordance (93.5%). For *Staphylococcus*, one phylogeny showed no significant difference and showed the highest concordance (100%). For *Legionella*, three phylogenies showed no significant difference. Concordance for these phylogenies was very high, ranking joint first, 12th, and 22nd out of 408 loci (88.9%, 82.2%, and 80.0% concordance respectively).

Hypervariable regions of the 16S rRNA gene have grown increasingly popular in phylogenetics, in species delineation, and more recently in microbiome studies [49–56]. Some studies have suggested that these hypervariable regions are able to distinguish between species with more accuracy than the full gene [57]. To evaluate these hypervariable regions, we extracted each region, constructed phylogenies, and calculated levels of concordance with the respective species phylogeny. Note, *Legionella* lacked the V9 region. V1-V2 were extracted together in a single alignment as these two regions are typically combined due to their combined length being suitable for Illumina sequencing. V3-V4 were extracted both individually and together as they are commonly combined for higher species delineation accuracy [58]. Overall, concordance for the hypervariable regions was lower than those for the full-length gene (Fig. 1). Specifically, the concordance for the full-length gene ranged from 1.8 to 5.0 times higher than the corresponding averages for the hypervariable regions. The region with the highest concordance was not consistent across the genera (Figs. 1 and S4). Specifically, the most concordant region for *Staphylococcus* and *Legionella* was V1-V2, whereas for *Campylobacter*, the V3-V4 and V5 regions were tied, and for *Clostridium*, the V3-V4 and V4 regions were tied. On average, the V3-V4 region showed the most concordance (Fig. S4).

The accuracy of a phylogeny hinges greatly on the underlying nucleotide alignment, and numerous approaches have been developed to identify and mask regions of an alignment judged to have a weak phylogenetic signal. Many of these approaches are based on the assumption that highly variable regions have a weak or unreliable signal. Specifically, high variability (nucleotide diversity) is assumed to be the product of an elevated mutation rate, which may result in the region becoming substitution saturated, which may confound a phylogeny due to underestimation of genetic distances. One approach to measuring this variability is to calculate entropy or information content for alignment columns. In an attempt to improve the phylogenetic signal, columns that exceed a pre-determined threshold are then masked. To explore the effectiveness of this approach for the 16S rRNA gene, we determined the level of concordance with the species phylogeny for alignments for each genus where the top 10% most entropic alignment columns were masked. This approach decreased concordance for *Clostridium*,* Staphylococcus*, and *Legionella* (Table S4A-D). Specifically, concordance was between 1.5 and 3.3 times higher for the unmasked gene. *Campylobacter* was the exception, here concordance when masked remained the same as the un-masked concordance. We explored additional masking levels of 20% and 30%; however, at 20%, only 70% of the gene’s alignment remained and at 30% the entire gene was masked. Additionally, we determined levels of concordance for the V3 region, V4 region, and V3-V4 regions combined when entropy masked at the 10% level. The V3 and V3-V4 regions for *Staphylococcus* could only be masked at the 5% level due to low entropy. When the V3, V4, and V3-V4 regions were masked, they consistently suffered a decrease in concordance (Tables S4A-D). Concordance prior to masking ranged from 1.3 to 8.0 times as high and concordance for two regions (V4 — *Staphylococcus* and V3 — *Campylobacter*) reduced to zero.

**Fig. 2:**
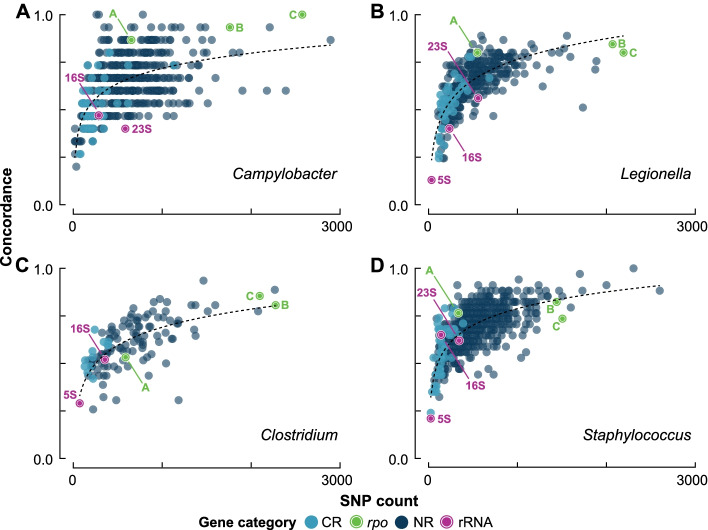
Levels of concordance with the species phylogeny for core genes plotted against each gene’s SNP count. For each genus, the logarithmic model is shown. Gene and gene category labels and coloring follow Fig. 1

A key factor affecting the amount of phylogenetic information within a gene alignment is the number of SNPs. Concordantly, we found that the level of concordance for any given gene was strongly predicted by alignment SNP count (Fig. 2). For example, the average number of SNPs for the 16S rRNA gene was less than half that of non-ribosomal genes (254 and 604 respectively), which reflects the gene’s relatively poor concordance. To explore the relationship between gene SNP count and concordance, we plotted the number of SNPs within each core gene alignment against its concordance (Fig. 2). Visual inspection of the plots suggested that both logarithmic and logistic regression models may be appropriate for these data. Results of a fivefold cross-validation procedure [59] indicated that the logarithmic model was preferred (Table S6). Figure 2 shows that as the number of SNPs in a gene’s alignment increases, there is a rapid initial increase in concordance becoming more plateaued after 500–1000 SNPs. To explore this observation further, we concatenated the five gene alignments with the lowest concordance as well as the ten gene alignments with the lowest concordance and produced new phylogenies and levels of concordance. Comparing the average concordance of the five lowest scoring genes to the concordance of their concatenation (Table S7), the level increased from 26 to 64% (average 142 SNPs to 712 SNPs) in *Legionella*, from 32 to 68% (70 to 352 SNPs) in *Staphylococcus*, from 32 to 40% (512 to 2562 SNPs) in *Clostridium*, and from 27 to 67% (141 to 707 SNPs) in *Campylobacter*. With the exception of *Campylobacter*, concordance for the ten gene concatenation had a greater increase. Specifically, the level increased from 29 to 76% (average 145 SNPs to 1448 SNPs) for *Legionella*, from 36 to 74% (77 to 768 SNPs) for *Staphylococcus*, and from 37 to 47% (602 to 3800 SNPs) for *Clostridium*. Although the concordance for *Campylobacter* did increase (30 to 60%, 244 to 2443 SNPs), the increase was slightly lower than that obtained for the five gene concatenation (67%). The increase in SNPs for each genus was closely matched by an increase in gene alignment length (Table S4A-D). To compare the effect of SNP count and gene length on levels of concordance further, for each genus, we compared the sum-squared error around a logarithmic model for plots of SNP count vs concordance (Fig. 2) and gene length vs concordance (Fig. S5). We found all values to be lower for SNP count, suggesting a stronger relationship (Table S8). Variation in nucleotide mutation rate among genes is likely an important factor affecting the relationship between gene length and concordance as genes with similar or identical SNP counts could have different lengths and vice versa.

**Fig. 3:**
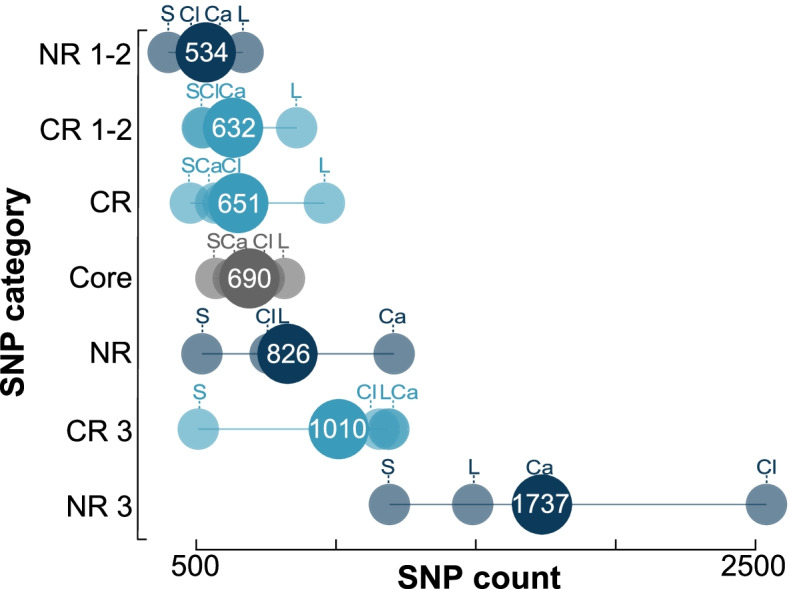
Dot plot showing the number of SNPs required for 80% concordance with the species phylogeny for seven SNP categories (see text). Non-ribosomal SNPs (NR, dark blue), coding ribosomal SNPs (CR, light blue), core gene SNPs (Core, grey), 3rd and 1st/2.nd nucleotide positions from non-ribosomal genes (NR 3, NR 1–2; dark blue) and coding ribosomal genes (CR 3, CR 1–2; light blue). Genus labels follow Fig. 1. The average number of SNPs necessary for 80% concordance for each SNP category is indicated by larger dots

**Fig. 4:**
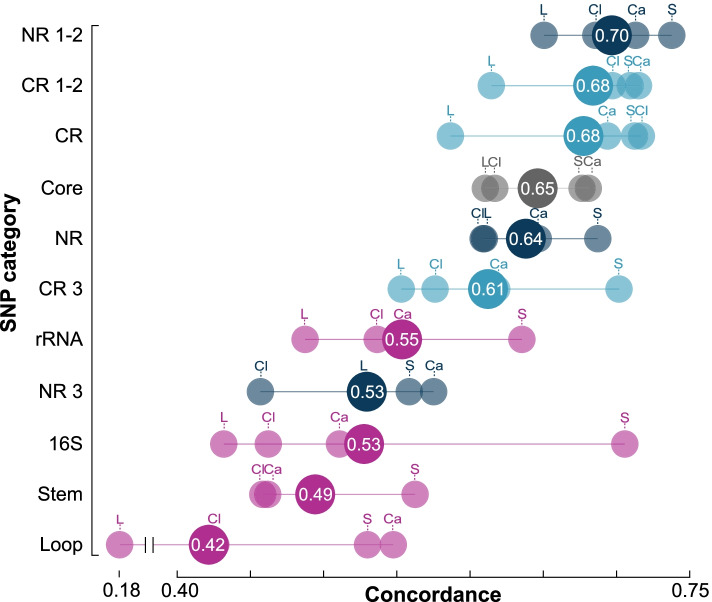
For each genus and SNP category, a dot plot showing levels of concordance predicted using the best fit logarithmic equation where the *y*-value was the average SNP count for rRNA alignments (266 nt) (see text for rationale). Large dots show average concordance for each genus. Non-ribosomal SNPs (NR) are shown in dark blue, coding ribosomal SNPs (CR) in light blue, core SNPs in grey, and rRNA SNPs in pink. Genus labels follow Fig. 1

In addition to alignment SNP count, we also found that the type of SNP influenced concordance. SNPs can be categorized by factors that affect their substation rate, which, if accelerated, can confound the phylogenetic signal due to substitution saturation. Multiple factors can affect the rate, such as structure and function of the gene product and differences between nucleotide position; for example, position within a codon for protein coding genes and whether the nucleotide is paired or un-paired within the secondary structure of rRNA genes. Paired nucleotides are connected via hydrogen bonds and form stems whereas un-paired nucleotides form loops. To explore these factors, we delineated eleven SNP categories as follows: (1) non-ribosomal [NR]; (2) non-ribosomal 1st and 2nd codon position [NR 1–2]; (3) non-ribosomal 3rd codon position [NR 3]; (4) protein coding ribosomal [CR]; (5) protein coding ribosomal 1st and 2nd codon position [CR 1–2]; (6) protein coding ribosomal 3rd codon position [CR 3]; (7) 16S, 23S, and 5S rRNA combined [rRNA]; (8) only 16S rRNA; (9) 16S rRNA paired “stem” nucleotide; (10) 16S rRNA un-paired “loop” nucleotide; and (11) any category of SNP from a single-copy core gene [core]. For all SNP categories, a fivefold cross-validation again showed that a logarithmic model best described the relationship between concordance and SNP count (Fig. S6, Table S6). Applying this model to the core SNP category, we found that the number of SNPs required to produce a phylogeny with 80% concordance ranged from 570 (*Staphylococcus*) to 816 (*Legionella*) with an overall average of 690 (Fig. 3, Table S9). For the remaining SNP categories (excluding rRNA) and averaging across the genera, we found that 1st and 2nd nucleotide position SNPs from non-ribosomal genes [NR 1–2] required the fewest number of SNPs for 80% concordance (534) (Fig. 3), followed by 1st and 2nd nucleotide position from coding ribosomal genes [CR 1–2] (632), then coding ribosomal genes [CR] (651), non-ribosomal genes [NR] (826), 3rd nucleotide position from coding ribosomal genes [CR 3] (1,010), and finally the 3rd nucleotide position from non-ribosomal genes [NR 3] (1,737). The four rRNA categories (all rRNA genes, 16S rRNA, 16S rRNA stem, and 16S rRNA loop) fell below our threshold of 1000 SNPs (see “Methods”). Therefore, we additionally compared all eleven categories based on what level of concordance would be produced using the average number of SNPs for the rRNA categories (266) (Fig. 4, Table S10)**.** The same ranking observed when using 1000 SNPs was observed for the non-rRNA categories, with NR 1–2 having the highest concordance at 69.7%. For the rRNA categories, the rRNA gene SNP category “rRNA” had the highest concordance (55.4%), followed by the 16S rRNA gene (52.8%), stem (49.4%), and lastly loop (42.1%) (Fig. 4).

### Phylogenetic concordance and gene biochemical characteristic

To determine if there was an association between concordance with the species phylogeny and any gene biochemical characteristic, core genes were annotated with Gene Ontology (GO) terms. To facilitate comparison across all four genera, we identified terms that were assigned to one or more genes in all four genera and designated them universal terms. We identified 75 universal GO terms. Overall, terms assigned to coding ribosomal [CR] genes had among the lowest concordance (Fig. S7). For example, CR genes were distributed among 11 terms and seven of these terms were among the bottom ten, with four having the lowest concordances of all terms. In order of lowest concordance first, these terms were structural constituent of ribosome, translation, ribosome, large ribosomal subunit, intracellular, ribosome biogenesis, small ribosomal subunit, RNA binding, methyltransferase activity, nucleic acid binding, and transferase activity. Figure S7 again shows a correlation between concordance and SNP count. For example, the terms assigned to CR genes had among the lowest SNP count. However, two terms (transferase activity and nucleic acid binding) had relatively high concordance and correspondingly high SNP count. In contrast to the pattern seen for terms assigned to CR genes, the terms assigned to *rpo* genes had among the highest concordance: specifically, DNA-directed RNA polymerase activity (ranked first), DNA-dependent transcription (ranked third), and DNA binding (ranked 13th).

### Inter-genus phylogenetic concordance and recombination/HGT

Given that the 16S rRNA gene and its hypervariable regions are often used in comparisons above the species level, we extended our pipeline to evaluate phylogenetic concordance with the species phylogeny at the inter-genus level. For this analysis, we elected to build a species phylogeny that was representative of the human gut microbiome — a diverse community spanning six phyla. We followed Liu et al. (2021) who delineated 54 core genera for this microbiome (31 families, 17 orders, and 12 classes) (Tables S11 and S12) [60]. To maximize the number of core genes, we elected to use only complete genome sequences available at NCBI. Using two representative species for each genus, we recovered 82 species representing 41 of the 54 core genera. Although genome sequences were unavailable for 13 of the core genera (Table S12), we were able to capture all families. Strain information and assembly IDs for which genome sequences were available for our analysis are presented in Table S11**.**

**Fig. 5:**
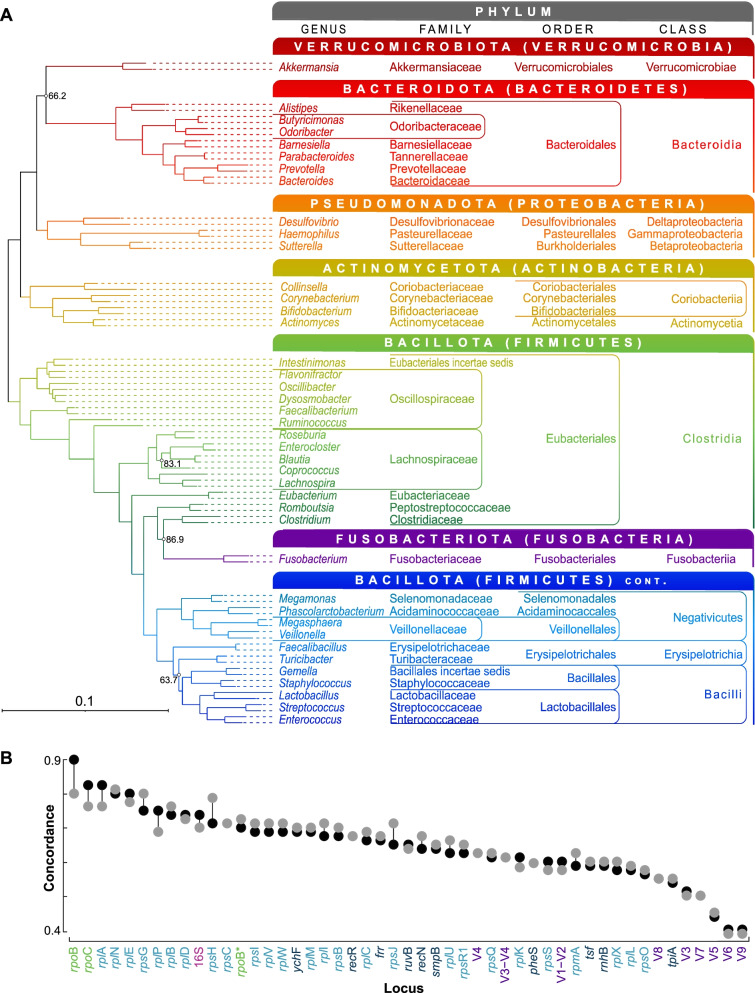
**A** ML phylogeny showing relationship among 82 species that represent 41 core genera of the human gut microbiome. Taxonomic nomenclature and classification follow NCBI, and for each phylum, updated names are shown with longstanding informal names shown in parentheses. Levels of bootstrap support lower than 90% are shown (500 replicates). For each genus, two representative species are included (names not shown — see Table S11 and Fig. S8A for details). **B** Dot plot showing levels of concordance for core genes and 16S hypervariable regions. Black dots show concordance with the species phylogeny and grey dots show concordance with the phylogeny representing a consensus of the topologies of each single-copy core gene phylogeny. The 440-bp section of the *rpo*B gene referred to in the text is shown with an asterisk. Gene and gene category labels and coloring follow Fig. 1

Following the same procedure used at the intra-genus level, we first used homologous gene clustering to delineate the core genome and identified 38 single-copy core genes (Table S13). Of these, four showed evidence of recombination/HGT and were excluded. The remaining 34 genes produced a species phylogeny that possessed strong bootstrap support (Figs. 5A and S8A) and showed good concordance with the consensus phylogeny differing by 11 bipartitions (86.3% concordance) (Fig. S8B). The bipartition differences were distributed evenly through the phylogeny and again involved minor rearrangements among closely placed taxa. To further examine the level of concordance between the two approaches, we examined how concordance was distributed among the separate gene phylogeny comparisons (i.e., comparison to the species phylogeny and comparison to the consensus phylogeny) and found this distribution to be highly concordant between the two approaches (Fig. 5B and Table S13).

A notable positioning in the species phylogeny was the placement of Fusobacterium. Historically, this genus belongs to the phylum Fusobacteriota (formerly Fusobacteria); however, in our phylogeny, Fusobacterium fell within the phylum Bacillota (formally Firmicutes). Phylogenetic instability of Fusobacteriota and placement within Bacillota has been reported previously [61, 62]. To explore this placement further, we examined all 38 gene phylogenies and found that all but three (16S, *rpo*C, and 50S L27) placed Fusobacterium within Bacillota. Of these three, two (16S and *rpo*C) tested positive for recombination. Inspection of the *rpo*C sequence alignment showed a ~ 600 bp insertion starting at nucleotide position 3083 that was shared among Fusobacterium, all taxa within the Bacteroidota, Pseudomonadota, and Verrucomicrobiota phyla, and all four genera of the Negativicutes class within Bacillota. A possible explanation for this insertion is an ancient recombination event involving these taxa. To explore this, we removed the insertion from the alignment and re-built the phylogeny. The resulting phylogeny placed Fusobacterium within Bacillota, supporting the recombination hypothesis. It is possible that similar recombination events may have confounded previous phylogenetic analyses involving Fusobacteriota.

Concordance with the species phylogeny for each gene, 16S rRNA hypervariable regions, and a ~ 440 bp section of *rpo*B (see “Discussion”) was evaluated and the full 16S rRNA gene ranked 10th out of 49, with 73.8% concordance (Fig. 5B and Table S13). Concordance for all loci ranged from 90.0 to 40.0%, with *rpo*B ranking highest and most hypervariable regions (V3, V5, V6, V7, V8, and V9) lowest (55.0–40.0%). Concordance for the remaining hypervariable regions (V4, V3-V4, and V1-V2) ranged from 62.5 to 60.0% (30th–36th). To again complement our gene ranking approach based on concordance with the species phylogeny, we calculated log-likelihood values for each gene phylogeny and ran the approximately unbiased (AU) topology test. Results again showed the likelihood values to be highly concordant with concordance levels (Table S13) and one phylogeny (*rpo*B) showed no significant difference.

## Discussion

### rRNA and protein coding ribosomal genes show weak concordance with the species phylogeny

Focusing first on our intra-genus analyses, we show a weak concordance with the species phylogeny for the 16S rRNA gene in four taxonomically diverse and clinically relevant genera: *Staphylococcus*,* Clostridium*,* Legionella*, and *Campylobacter*. One explanation for this weak concordance is recombination and HGT, which, concordant with accumulating reports in the literature [13–16, 63–65], we detected in all four genera. Another explanation is the gene’s low SNP count. The gene possessed an average of 254 SNPs, which was less than two-fifths of that required to produce a phylogeny 80% concordant with the species phylogeny. These findings are concordant with a recent study that showed stronger taxonomic resolution for the 16S-23S-5S operon compared to just the 16S rRNA gene [66]. Only 17.0% of 16S rRNA nucleotide sites were variable and this low SNP proportion likely reflects ribosomal functional constraint on nucleotide substitution for the gene’s RNA imposed by the fundamental translational processes of tRNA binding, mRNA decoding, and peptidyl transfer. Intrinsic to these processes is the tertiary structure of the rRNA molecule, which acts as a scaffold for the ribosomal proteins. The importance of this structure with regard to rRNA evolution was recently highlighted by Bernier et al. who showed that approximately 90% of rRNA forms a tertiary “common core” of elements described as helices, junctions, and loops that are highly conserved in ribosomes of all extant species [67]. This work is consistent with our finding that 83.0% of nucleotide sites for 16S rRNA across our four taxonomic groups showed no variation. In addition to the 16S rRNA gene as a whole, our SNP-by-SNP analysis showed low concordance for 16S rRNA SNPs and rRNA SNPs in general when compared to protein coding DNA SNPs. Numerous studies have shown that rRNA nucleotide substitution rate is highly conserved at the center of the ribosome and increases as you move to the surface [68–74]. These observations likely reflect the fact that nucleotides at the surface of the ribosome are distal from the active binding sites at the core and are therefore less important in translational processes and maintenance of the rRNA tertiary structure. Furthermore, given their relatively poor concordance (close to that of the third codon position in non-ribosomal genes), there appears to be a significant release of functional constraint on this small proportion of nucleotides.

Within the literature, there has been considerable focus on the secondary structure mutation rate for eukaryote rRNA. However, results are conflicting, with some studies showing a faster rate for stems, others for loops, and others showing no difference [74–80]. For prokaryotes, studies have suggested that stems evolve more rapidly than loops [72, 81]. It is proposed that these paired stem regions experience selective pressure to maintain the rRNA secondary structure. Specifically, structure-disrupting mutations are compensated for via positive selection of a secondary mutation, which restores the secondary structure of the molecule [82–88]. A possible ramification of an accelerated mutation rate skewed towards stems may be increased substitution saturation and reduced phylogenetic signal. However, here, we show for a diverse range of taxa that the number of SNPs is distributed evenly among stems and loops and concordance is lower for loops suggesting that un-paired nucleotides may be more susceptible to saturation (Fig. 4).

Although concordance for protein coding ribosomal genes [CR] was higher than for rRNA genes, it was still lower than for non-ribosomal genes [NR]. One explanation for this weaker concordance is recombination/HGT, as 43.0% of protein coding ribosomal genes exhibited evidence of this through at least one of the tests. However, this number was relatively low when compared to rRNA genes (83.0%) and very similar to non-ribosomal genes (40.0%) (Table S5). Additional factors may be the relatively low average SNP count and proportion per gene when compared to non-ribosomal genes. Specifically, the SNP count was approximately one-third (218 vs 604) and the SNP proportion was 50.4% as opposed to 66.0%. (Table S5 and Fig. S9). A key factor governing this lower number of SNPs was a relatively short average gene length (434 bp) — approximately half that of non-ribosomal genes (928 bp) (Fig. S5). This short gene, and therefore protein length, likely facilitates rapid ribosome production and assembly that is required during periods of high metabolic activity and cell division.

Our analysis to associate species phylogeny concordance with gene biochemical characteristic confirmed a poor concordance for genes involved in translation. In contrast, genes involved in transcription (an equally fundamental, yet distinct cellular process) possessed one of the strongest levels of concordance (Fig. S7). Specifically, the concordance for coding ribosomal genes [CR] was 53.5% as opposed to 81.3% for *rpo* genes (Table S5). This difference is most likely due to the much longer length (and hence SNP count) for RNA polymerase genes — average 2895 bp vs 434 bp (Table S5). Despite the large difference in size for these gene types, their average SNP proportions were very similar and relatively low (*rpo*ABC = 52.4% vs CR = 50.4%) (Fig. S9 and Table S5). Proteins with slow mutation rates typically form large complexes and have surfaces that interact with other proteins, which results in elevated selection pressure to maintain function and prevent misfolding or aggregation [89–91]. RNA polymerase proteins and in particular ribosomal proteins exemplify these characteristics [92–96] likely contributing to the low SNP proportions we detected. Moreover, recent cryo-EM studies have suggested that the bacterial ribosome and RNA polymerase form a transcription-translation complex during coupling of transcription and translation [94, 97–101]. In addition, these gene categories have above average expression levels [102, 103], which has been correlated with slow mutation rates and is thought to contribute to the selection pressure described above [91].

In contrast to rRNA SNPs, our SNP-by-SNP analysis showed that protein coding ribosomal SNPs possessed a level of concordance roughly equivalent to that of non-ribosomal genes (concordance for first and second codon position, third codon position, and all codon positions had similar distributions — Fig. 4). Consequently, there appears to be sufficient functional constraint to limit substitution saturation and preserve the phylogenetic signal. These findings validate a phylogenetic approach where multiple coding ribosomal genes are combined, with our results indicating that three or more genes would be sufficient. This approach would be practical when attempting to infer phylogenetic relationships over wide evolutionary distances.

### 16S rRNA hypervariable regions show weak concordance with the species phylogeny: implications for microbiome studies

Concordance with the species phylogeny for the hypervariable regions of the 16S rRNA gene was typically lower than that of the whole gene (a few regions were comparable) and masking provided little to no benefit (Fig. 1 and Tables S4A-D). Again, a major factor likely contributing to these findings was low SNP count. The average number of SNPs for the hypervariable regions was 36, approximately one-nineteenth the number of SNPs necessary for 80% concordance. Although entropy masking has the potential to improve the phylogenetic signal by masking sites that may be substitution saturated, the trade-off is a decrease in the number of SNPs from which to derive phylogenetic information. For the full 16S rRNA gene, masking decreased the average number of SNPs from 254 to 137 — approximately one-fifth the number necessary for 80% concordance. Masking the V3, V4, and V3-4 regions decreased the SNP count and concordance even further. Our results highlight how concordance with the species phylogeny for different genes changes depending on the evolutionary scale [104, 105]. Moving from intra- to inter-genus level for 16S rRNA increased the number of SNPs threefold with an accompanying increase in concordance with the species phylogeny. In contrast to the SNPs observed at the intra-genus level, the SNPs acquired at the inter-genus level show much improved concordance with the species phylogeny, suggesting a slower rate of evolution and minimal saturation, which in turn suggests that these nucleotides may be closer to the core of the ribosome.

Hypervariable regions of the 16S rRNA gene are regularly used in microbiome studies and diversity metrics are often calculated using approaches that incorporate phylogenetic information: for example, UniFrac for beta diversity and Faith’s phylogenetic diversity for alpha diversity. Specifically, both approaches require a phylogeny whose patristic distances (branch lengths) among OTUs are used to calculate the metric. Statistically significant differences in community composition may hinge on subtle differences in beta diversity and a poor phylogenetic signal may confound these analyses with possibly important ramifications — for example, studies of the human microbiome as it pertains to health and disease. Our results demonstrate that if taxonomic assignments at the intra-genus level are employed, the use of diversity metrics that incorporate phylogenetic signal when using the 16S rRNA gene and any of its hypervariable regions are problematic and should be discouraged. In addition, we show that entropy masking does not resolve the problem, rather it further decreases concordance and likewise should be discouraged. Another problem with 16S rRNA gene hypervariable regions is that they often fail to distinguish taxa below the genus level [5]. Lastly, it is also important to consider the large variation in 16S rRNA gene copy number among bacterial genomes as this has a strong potential to skew taxon frequency measurements and diversity metrics (Fig. S3).

Although no single gene has the appropriate combination of conserved and hypervariable regions to replicate the 16S rRNA gene’s ability to capture all members of any microbial community, other genes may be utilized to provide more targeted (narrow) taxonomic profiles that have higher taxonomic resolution and are more accurate and reliable [47, 106]. A good example is the *rpo*B gene (β subunit of RNA polymerase). This gene (along with *rpo*C — β’ subunit) has several beneficial characteristics. It is long, contains conserved and hypervariable regions, exists universally as a single-copy gene in bacteria [107], and shows high concordance (85.0% average at the intra-genus level and 90.0% at the inter-genus level). Unfortunately, the *rpo*B gene is too variable across all bacteria to facilitate the design of universal PCR primers. However, various sections of *rpo*B have been used (often paired with 16S rRNA) to profile select members of a community [27]. For example, a Web of Science key word search detected 148 studies utilizing *rpo*B to profile microbial communities. Numerous studies have targeted the same general region of the gene [47, 106, 108–110] and the recent primer pair of Ogier et al. [47] (~ 440 bp) targeting the nematode gut microbiome captures this region. We evaluated concordance for this region and measured an average of 47.6% at the intra-genus level and 70.0% at the inter-genus level (Table S4A-D and S13). While this concordance is markedly lower than that of the full *rpo*B gene, at the intra-genus level, it is considerably higher than the average level of concordance for the 16S rRNA hypervariable regions (19.8%) and the masked full 16S rRNA gene (31.8%). At the inter-genus level, however, the concordance is more comparable [70.0% (partial *rpo*B) vs 72.8% (full 16S rRNA gene)]. Given that concordance for the full 16S rRNA gene is much improved at the inter-genus level, a robust strategy might be to pair it with a more targeted locus (the hypervariable regions still suffer from low concordance and their use is not recommended). The full 16S rRNA gene would provide information on bulk changes at and above the inter-genus level across all bacteria and one or more additional loci would provide targeted intra-genus level information. Improvements in long read sequencing technology such as PacBio now make whole gene sequencing at the community level more feasible. However, it should be understood that the 16S rRNA gene is still prone to recombination and phylogenetic error. Furthermore, variation in copy number among strains and species still has the potential to skew diversity metrics.

## Conclusion

In summary, 16S rRNA gene nucleotide substitution at the intra-genus level is limited to a small proportion of the gene that appears localized at the surface of the ribosome where functional constraint is released. These factors result in a gene with a low number of intra-genus level SNPs that likely experience substitution saturation. Coupled with recombination and HGT, these factors combine to produce a gene with one of the weakest levels of concordance with the species phylogeny at this taxonomic level of any gene in the core genome. Consequently, we advocate discontinuing its use in species delineation and phylogenetics and recommend utilizing whole genome sequences or multiple coding ribosomal gene sequences where possible. Concordance with the species phylogeny for the hypervariable regions of the 16S rRNA gene at the intra-genus level is weaker still and entropy masking only exacerbates the situation. At the inter-genus level, although concordance for the whole 16S rRNA gene is much improved, the hypervariable regions still show relatively low concordance. These findings coupled with those showing recombination/HGT and high variation in copy number have important ramifications for microbial community studies where these regions are used extensively. Specifically, their use could be misleading; in particular, if they are the sole locus employed and we recommend alternative approaches where possible. For example, whole genome metagenomics is a powerful approach that attempts to assemble all genomes within the community. Recent progress with long read sequencing technology has made this approach more feasible by lowering the complexity of genome assembly — a serious impediment for short read technology. However, whole genome metagenomics (short or long read technology) is technically challenging and still cost prohibitive in many situations. Consequently, amplicon or gene sequencing remains an effective and practical approach for many microbial community studies. Therefore, despite the limitations, and depending on the taxonomic capture required, a practical compromise could be full 16S rRNA gene sequencing coupled with additional more taxonomically targeted loci. Regardless, widely used analytical approaches that incorporate phylogenetic information into the calculation of diversity metrics have the potential to confound results when using 16S rRNA gene sequence and are strongly discouraged.

## Methods

### Sequence data annotation

For each strain genome sequence (contigs and complete genomes), open reading frames (ORFs) were determined using Prokka v1.11 [111] and all genomes were reannotated using custom annotation databases for each genus using Prokka v1.11. These custom annotation databases included the most current annotations available at RefSeq genome for each of the four genera.

### Homologous gene clustering and phylogenetic analyses

Homologous gene clustering for each genus was performed using the Markov Clustering (MCL) algorithm, as implemented in the software MCLBlastLINE [112]. The software uses MCL to assign gene sequences to clusters with putative shared homology based on a BLASTp search between all pairs of protein sequences (E-value cutoff: 1e − 5). An inflation parameter of 1.8 was specified in the MCL algorithm [112] as simulations have shown this value to be generally robust to false positives and negatives [113]. Results of these analyses were used to build gene content tables, which provide information regarding the presence or absence of a gene sequence within a homologous gene cluster (as well as copy number). For each genus, MCL gene clusters were considered part of the core genome if they were present in all genomes. We excluded clusters containing paralogous genes by only selecting clusters that contained a single gene/genome (single-copy core clusters).

Nucleotide sequences for each single-copy core cluster were aligned using Probalign v1.4 [114]. The number of SNPs present in nucleotide alignments was determined using the BioPerl module Bio::PopGen::Statistics available from CPANM [115]. Each alignment was tested recombination using the Pairwise Homoplasy Index approach as implemented in the software PHI, which is part of the package PhiPack [44] and the single breakpoint (SBP) approach [45] as implemented in HyPhy [116]. The alignments were additionally tested for HGT using the software HGTector [46]. The PHI recombination test measures the significance of discordant phylogenies across sites in an alignment and is based on the compatibility of parsimoniously informative sites. The SBP recombination approach scans an alignment for possible break points that would be the result of recombination. Maximum likelihood (ML) phylogenies are built for each alignment segment on either side of the possible break point. Using this approach, alignments are considered putatively recombinant if they possess a single breakpoint with discordant phylogenies on either side. HGTector assesses protein sequences for HGT using a BLAST-based approach at NCBI. Sequences are flagged as horizontally acquired if the top BLAST hits for the gene are from user-defined distantly related species.

Using the generalized time-reversible (GTR) model of nucleotide substitution, an un-rooted ML phylogeny (gene phylogeny) for each alignment was produced using PhyML v3.0 [117]. The proportion of invariable sites and gamma shape parameter distribution were estimated using maximum likelihood. Branch support for these phylogenies was provided via 200 bootstrap replicates. Using the same approach with PhyML, the ML species phylogeny was constructed using a concatenation of all single-copy core gene cluster alignments that tested negative for all three recombination/HGT tests. Note that given our approach targets gene sequences (intact open reading frames) extracted from genome sequences, no intergenic nucleotide sequence is incorporated into the concatenation. The species phylogenies are presented in the supplementary material (Figs. S1A, C, E, and G) (intra-genus) and Figs. 5A and S8A (inter-genus). Branch support for these phylogenies was provided via 500 bootstrap replicates. Reliability of species phylogenies was further assessed by comparison to a second core gene phylogeny that represented a consensus of the ML topologies of each single-copy core gene phylogeny. The consensus phylogeny was constructed using the consense program within the PHYLIP package v3.6 [118]. We used the Majority Rule extended approach. Here, any grouping of taxa that occurs in more 50% of the phylogenies is included in the consensus phylogeny. For groupings that occur in less than 50% of the phylogenies, those that are compatible with the existing consensus topology are sequentially added based on their frequency of occurrence.

### Core gene phylogeny concordance with the species phylogeny

The topology of each single-copy core gene phylogeny was compared to its respective species phylogeny by constructing a consensus phylogeny between the two phylogenies using SumTrees (majority-rule) as implemented in Dendropy [119]. Concordance was quantified by calculating the proportion of concordant bipartitions and genes ranked accordingly. Our concordance metric equates to the Robinson-Foulds distance metric [120]. This metric is a quantification of discordant bipartitions. Here, we normalize the metric by expressing it as a proportion of concordant bipartitions. To complement our concordance ranking approach, we additionally calculated log-likelihood values for each gene phylogeny and ran the approximately unbiased (AU) topology test [48] using IQ-TREE v2.0.6 [121]. The AU test is a robust test that uses multi-scale bootstrapping of site-likelihoods to determine significant differences in topology.

### rRNA gene alignment, recombination/HGT analyses, and phylogenetic analyses

For the rRNA gene phylogenetic analyses, given the presence of multiple gene copies or the 16S rRNA, 23S rRNA, and 5S rRNA gene sequences within individual genomes, we randomly selected representative 16S rRNA, 23S rRNA, and 5S rRNA gene sequences for each species in each genus. For the recombination tests (PHI and SBP), we used all copies within a genome. rRNA gene sequences within genomes were located using BLASTn and aligned using the Fast Fourier Transform (MAFFT) v7.309 [122] plugins as implemented in Geneious v9.0.4 [123]. We were unable to test the rRNA genes for HGT using HGTector because the program requires an amino acid sequence as input. As an alternative, we took a phylogenetic approach. Using the rRNA alignments containing all gene copies, a maximum likelihood phylogeny was produced using the GTR model implemented in PhyML. A monophyletic grouping of gene copies indicated vertical inheritance, whereas a polyphyletic grouping provided evidence for HGT. For example, if species A possessed a horizontally exchanged copy of its 16S rRNA gene from species B, then we would expect the horizontally exchanged copy within species A to group among those from species B, separately from other vertically exchanged copies of the gene within species A.

### Phylogenetic concordance and nucleotide substitution

To assess the accuracy of logarithmic and logistic regression models to predict the relationship between concordance with the species phylogeny and the number of SNPs within a gene alignment, we used a fivefold cross-validation [124]. This analysis was done by randomly removing 20% of our data, fitting both the models based on the remaining 80% of our data, and then predicting the values of the removed datapoints based on the new fit. We then calculated the difference between the predicted values and the actual values for both models. Finally, we used the sum of the squared errors as the criterion to determine the best fit model.

To test if utilizing more genes, and thereby more SNPs, would increase concordance with the species phylogeny, we concatenated the alignments of the five and ten lowest scoring genes from each genus and produced two new ML phylogenies (procedure described above). We then compared these phylogenies to their respective species phylogeny to measure concordance. These measures were then compared to the average measures for the five and ten individual gene sets to determine if any increase in measurement of concordance occurred.

For each of the SNP categories, SNPs were extracted from the single-copy core gene alignments using a custom python script (see “Availability of data and materials”). Stem and loop nucleotides were determined by predicting the secondary structure using rPredictorDB [125]. rPredictorDB uses a database of experimentally derived rRNA secondary structures as a template to predict those of individual input sequences. Sequences in the 16S rRNA gene alignments were then cross referenced with their associated secondary structures and only SNPs that were identified as stems or loops in all species’ sequences were extracted.

For each genus and SNP category, we described the relationship between concordance with the species phylogeny and the number of SNPs. For seven of the eleven categories, the following procedure was followed (the four remaining categories were excluded due to a limited number of SNPs in their alignments — see below). SNP columns were incrementally extracted at random (without replication) from the core gene alignments, building 1000 separate alignments that ranged in size from 1 bp (SNP) to 1000 bp (SNPs). For each of these alignments, concordance was measured and plotted against the SNP count (Fig. S6). Finally, cross-validation was again used to determine the model that best described the relationship between concordance and SNP count. To compare the concordance among SNP categories, we determined the number of SNPs necessary for 80% concordance. This was accomplished as follows. Utilizing the best fit model for each category, the number of SNPs required was obtained by inverting the estimated model.

The four SNP categories with limited SNPs in their alignment were the rRNA categories (Table S14). With the exception of the stem category for *Legionella*, which contained only two SNPs, we extracted the maximum number of SNPs available in each alignment (64 through 871). We then followed the same iterative procedure described above building as many alignments as possible. Again, concordance was plotted against the SNP count and cross-validation used to determine the model that best described the relationship. To compare levels of concordance, we estimated concordance using the best fit model and the average number of SNPs for these categories (266). For comparative purposes, we applied the same procedure to the other seven categories and plotted the results (Fig. 4).

### Concordance with the species phylogeny and gene biochemical characteristic

Core genes were annotated with Gene Ontology (GO) terms [126] using InterProScan [127]. Terms that were assigned to one or more genes in all four genera were designated universal terms. For example, if the term “ATP binding” was only assigned to genes in three out of the four genera, it would not be considered universal. For each universal term, concordance for associated genes was averaged, both within each genus and across all genera. Terms were then ranked according to their concordance.

### Inter-genus phylogenetic concordance

Using the 82 closed genome sequences (species) outlined in the “Results” section, we used the same procedure used at the intra-genus level to evaluate phylogenetic concordance with the species phylogeny.

## Supplementary Information

**Additional file 1: Figure S1.** ML species and consensus phylogenies for each genus. **Figure S2.** rRNA HGT ML phylogenies for each genus. **Figure S3.** rRNA gene copy numbers for each species. **Figure S4.** 16S rRNA gene hypervariable region concordance for each genus. **Figure S5.** Gene length versus concordance for each genus. **Figure S6.** SNP count versus concordance for each SNP category and genus. **Figure S7.** Average level of concordance and SNP count for genes associated with universal GO terms. **Figure S8.** ML inter-genus phylogeny (includes individual species names). **Figure S9.** SNP proportion versus concordance for each SNP category.

**Additional file 2.** Excel spreadsheet including all supplementary tables, titles, and legends.

## Data Availability

The datasets supporting the conclusions of this article are included within the article and at 10.5281/zenodo.5976008.
