# Supplementary material for: Phylogenies of the 16S rRNA gene and its hypervariable regions lack concordance with core genome phylogenies
Source: Microbiome. 2022 Jul 8;10:104. doi: 10.1186/s40168-022-01295-y (PMC9264627; doi:10.1186/s40168-022-01295-y)
Supplement: Supplementary file 2 — Additional file 1: Figure S1. ML species and consensus phylogenies for each genus. Figure S2. rRNA HGT ML phylogenies for each genus. Figure S3. rRNA gene copy numbers for each species. Figure S4. 16S rRNA gene hypervariable region concordance for each genus. Figure S5. Gene length versus concordance for each genus. Figure S6. SNP count versus concordance for each SNP category and genus. Figure S7. Average level of concordance and SNP count for genes associated with universal GO terms. Figure S8. ML inter-genus phylogeny (includes individual species names). Figure S9. SNP proportion versus concordance for each SNP category. [file 40168_2022_1295_MOESM1_ESM.pdf]

Figure S1 A. *Campylobacter* species phylogeny

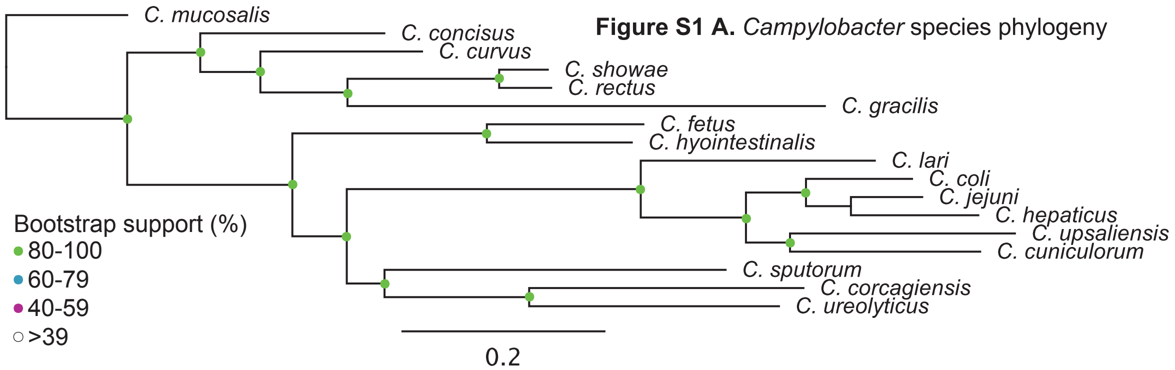

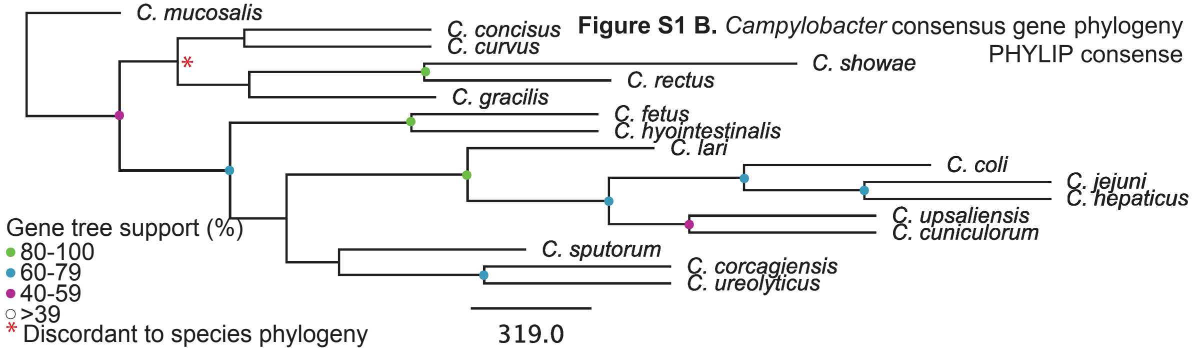

Figure S1 B. *Campylobacter* consensus gene phylogeny PHYLIP consense

Figure S1 C. *Clostridium* species phylogeny

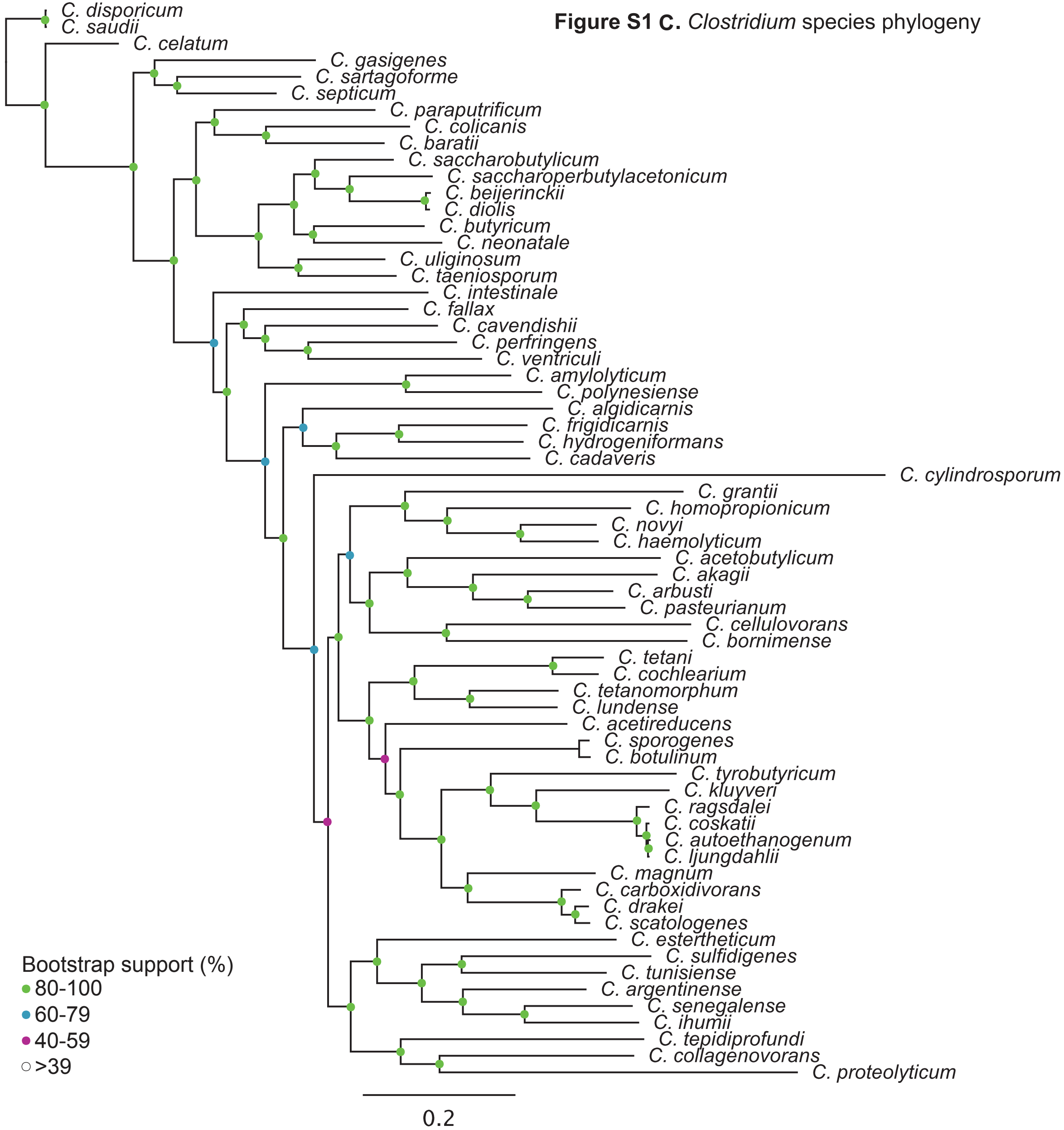

Figure S1 D. *Clostridium* consensus gene phylogeny
PHYLIP consense

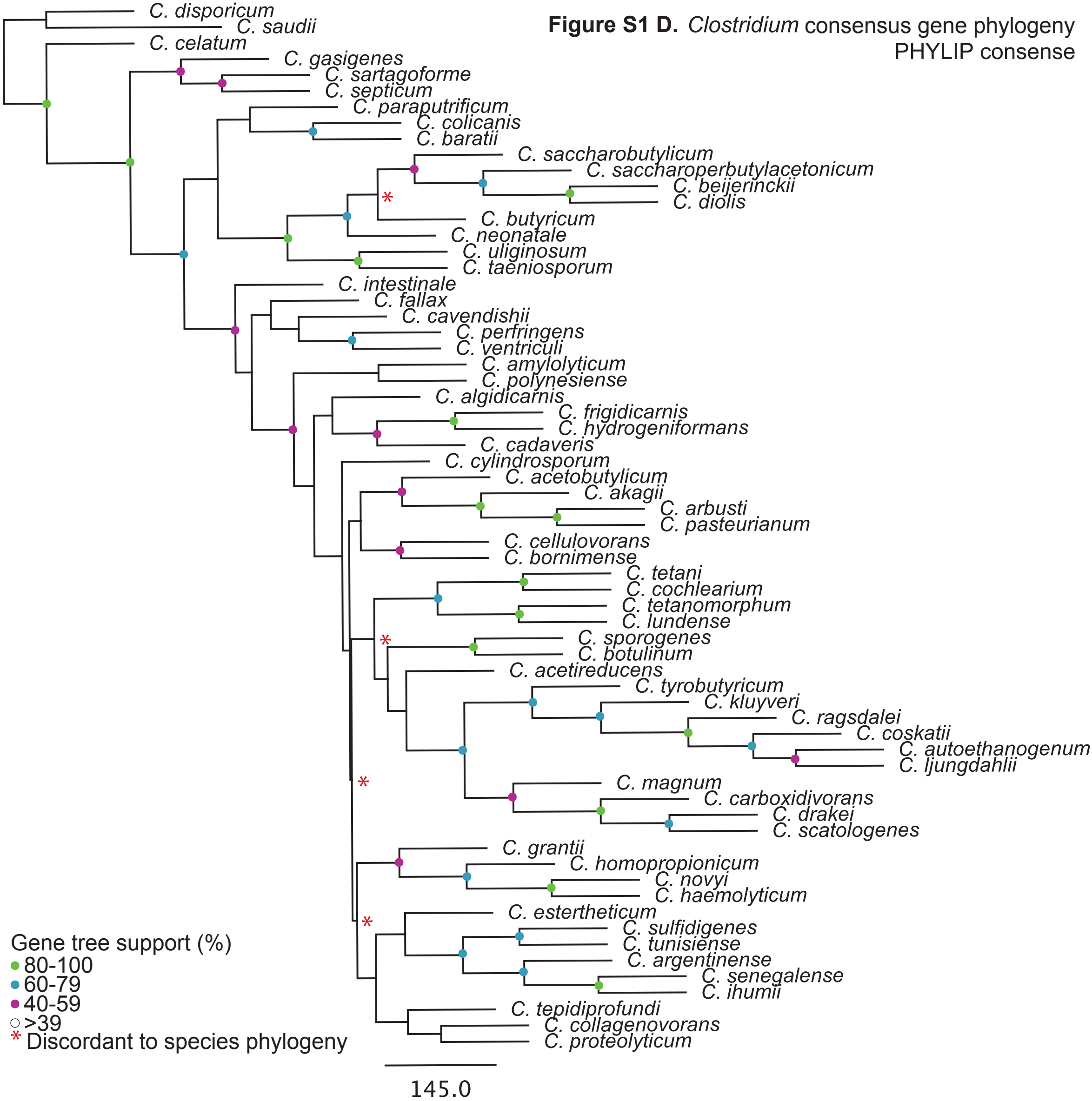

Figure S1 E. *Legionella* species phylogeny

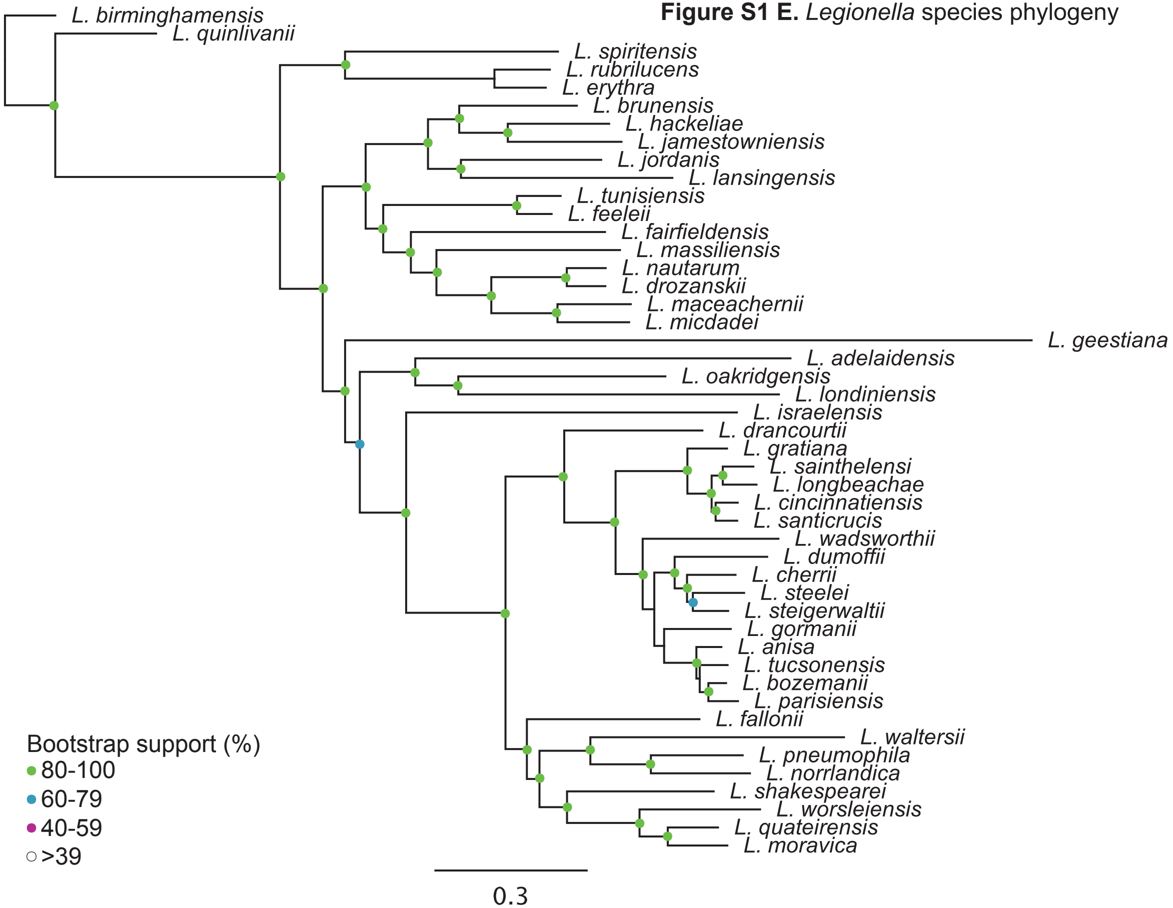

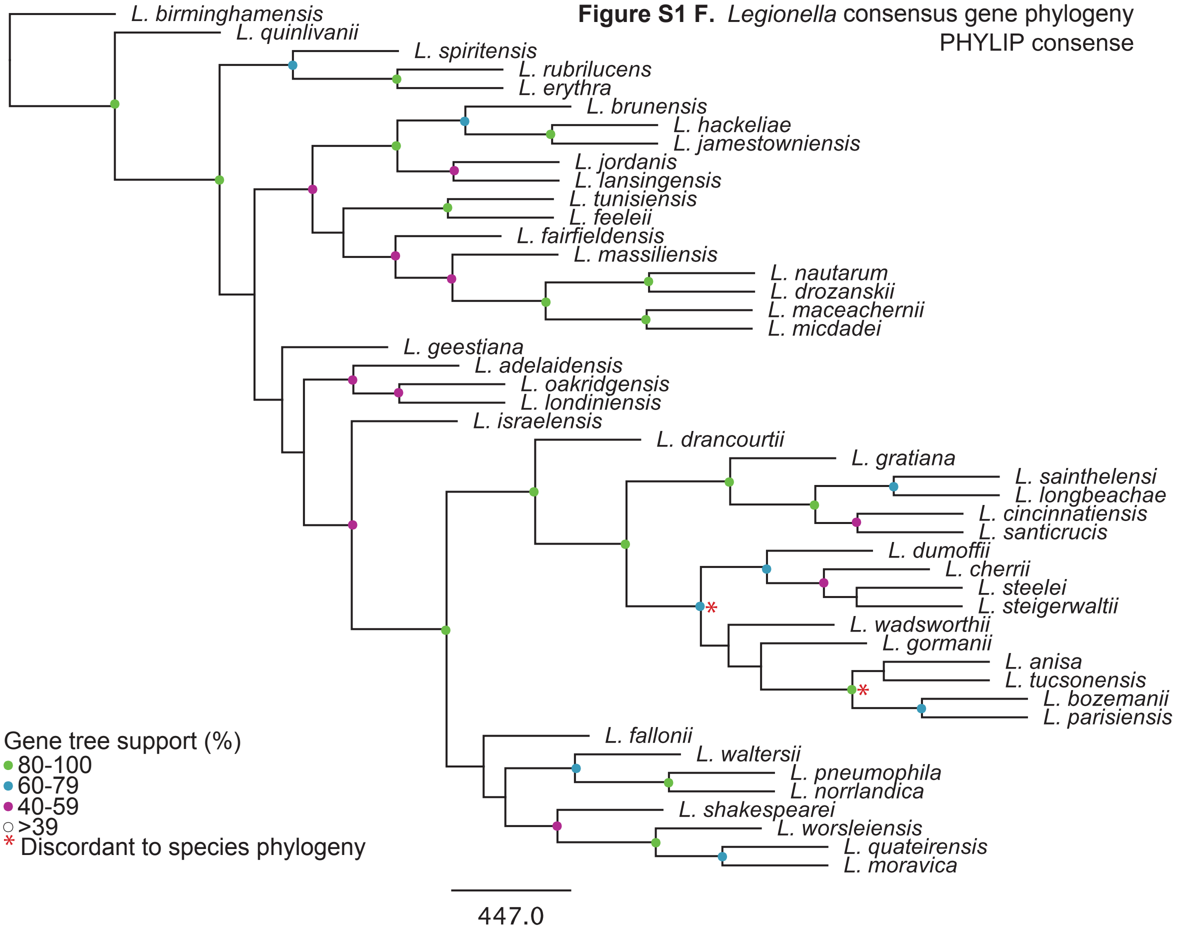

Figure S1 F. *Legionella* consensus gene phylogeny PHYLIP consense

Figure S1 G. *Staphylococcus* species phylogeny

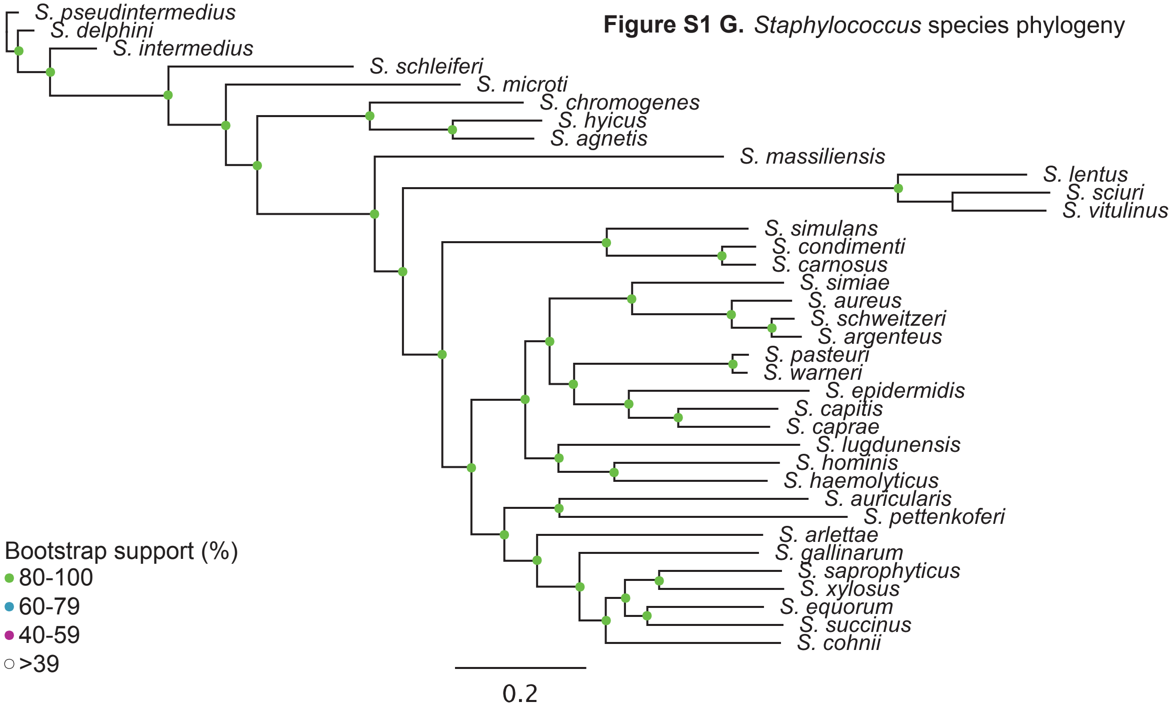

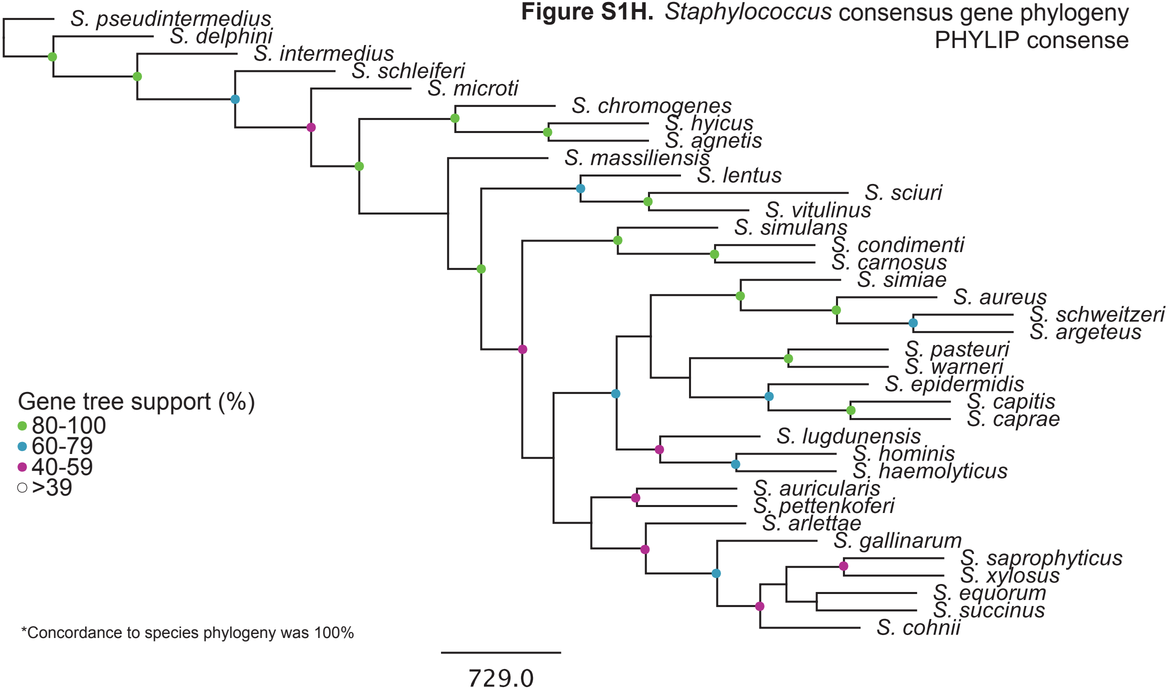

Figure S1H. *Staphylococcus* consensus gene phylogeny
PHYLIP consense

Figure S1. Concatenated maximum-likelihood and consensus species phylogenies for *Campylobacter, Clostridium, Legionella*, and *Staphylococcus*

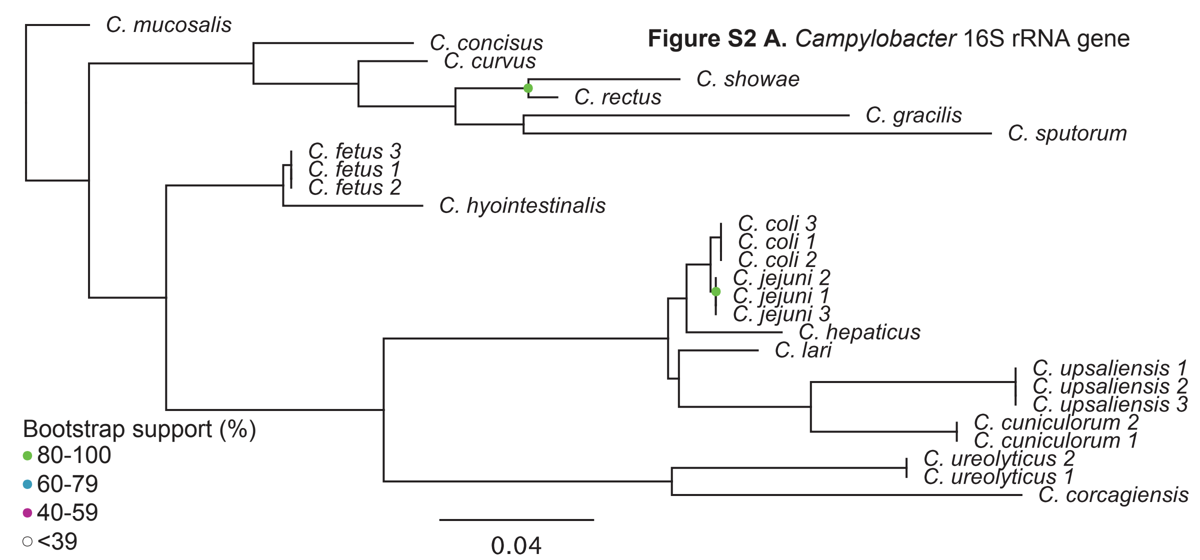

Figure S2 A. Campylobacter 16S rRNA gene
C. mucosalis
C. concisus
C. curvus
C. showae
C. rectus
C. gracilis
C. sputorum
C. fetus 3
C. fetus 1
C. fetus 2
C. hyointestinalis
C. coli 3
C. coli 1
C. coli 2
C. jejuni 2
C. jejuni 1
C. jejuni 3
C. hepaticus
C. lari
C. upsaliensis 1
C. upsaliensis 2
C. upsaliensis 3
C. cuniculorum 2
C. cuniculorum 1
C. ureolyticus 2
C. ureolyticus 1
C. corcagiensis
Bootstrap support (%)
80-100
60-79
40-59
<39
0.04

Figure S2 B. *Clostridium* 16S rRNA gene

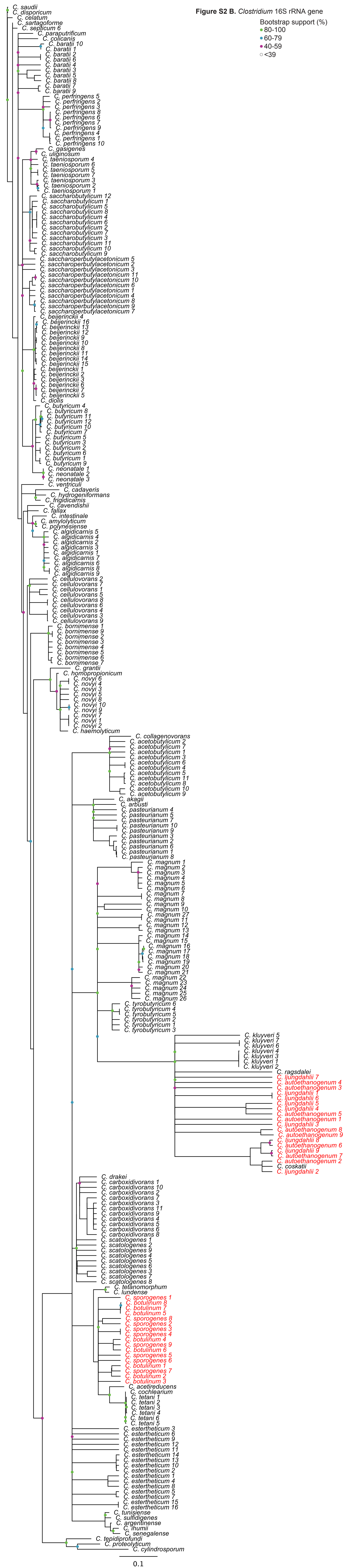

Figure S2 C. *Legionella* 16S rRNA gene

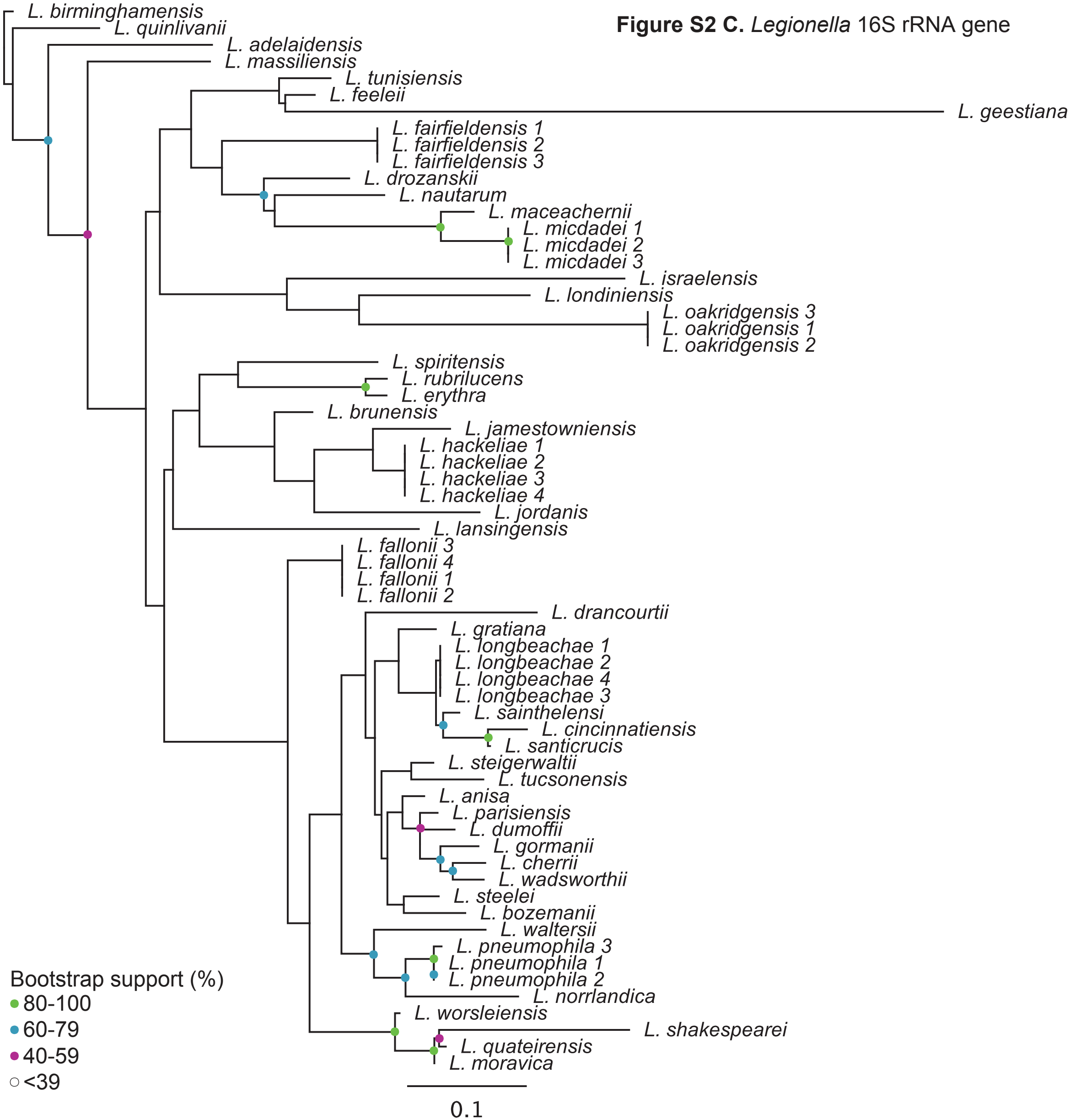

Figure S2 D. *Staphylococcus* 16S rRNA gene

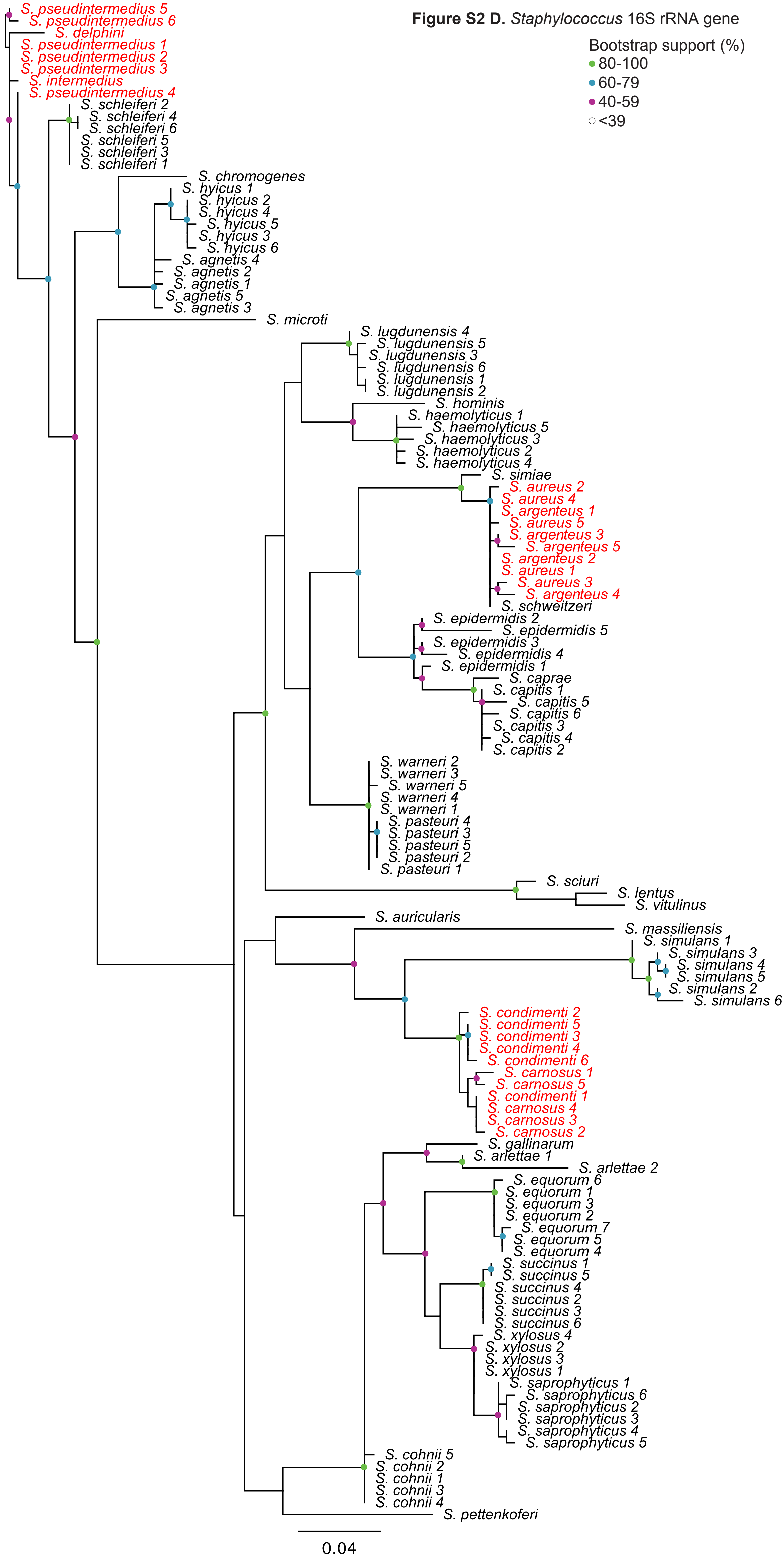

Figure S2 E. *Campylobacter* 23S rRNA gene

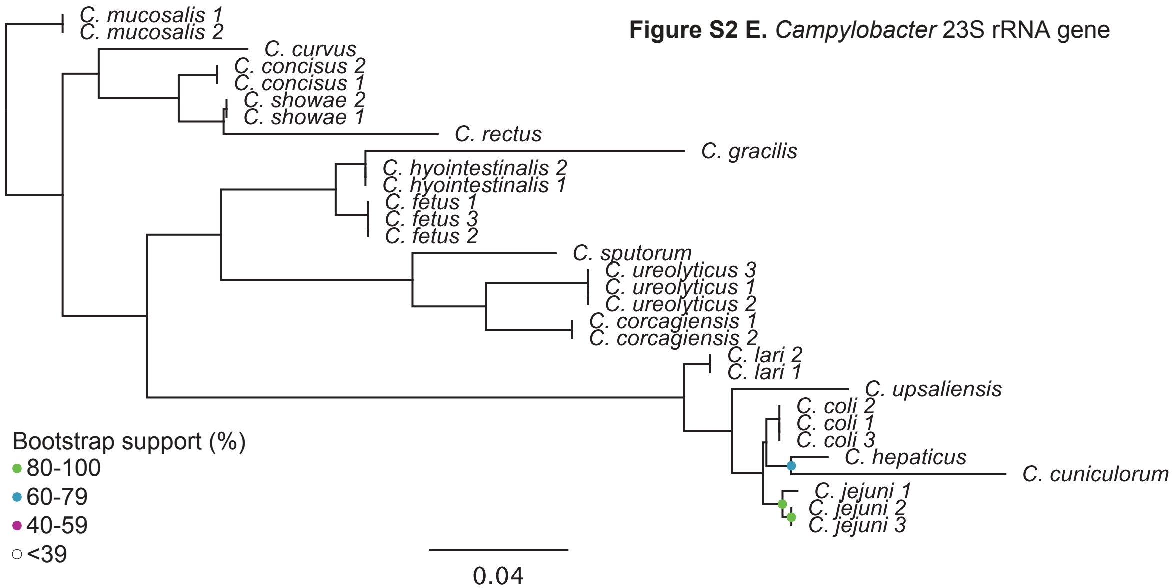

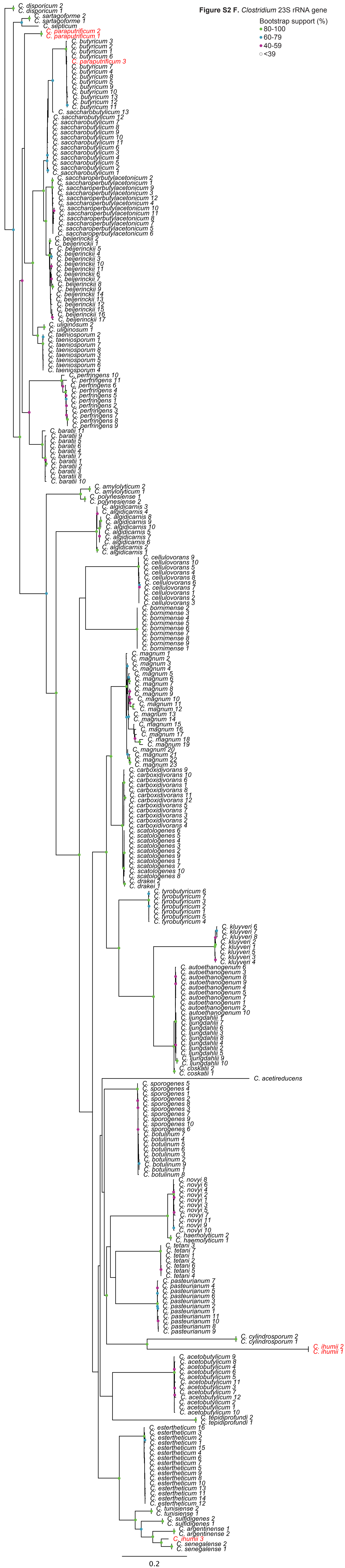

Figure S2 F. *Clostridium* 23S rRNA gene

Figure S2 G. *Legionella* 23S rRNA gene

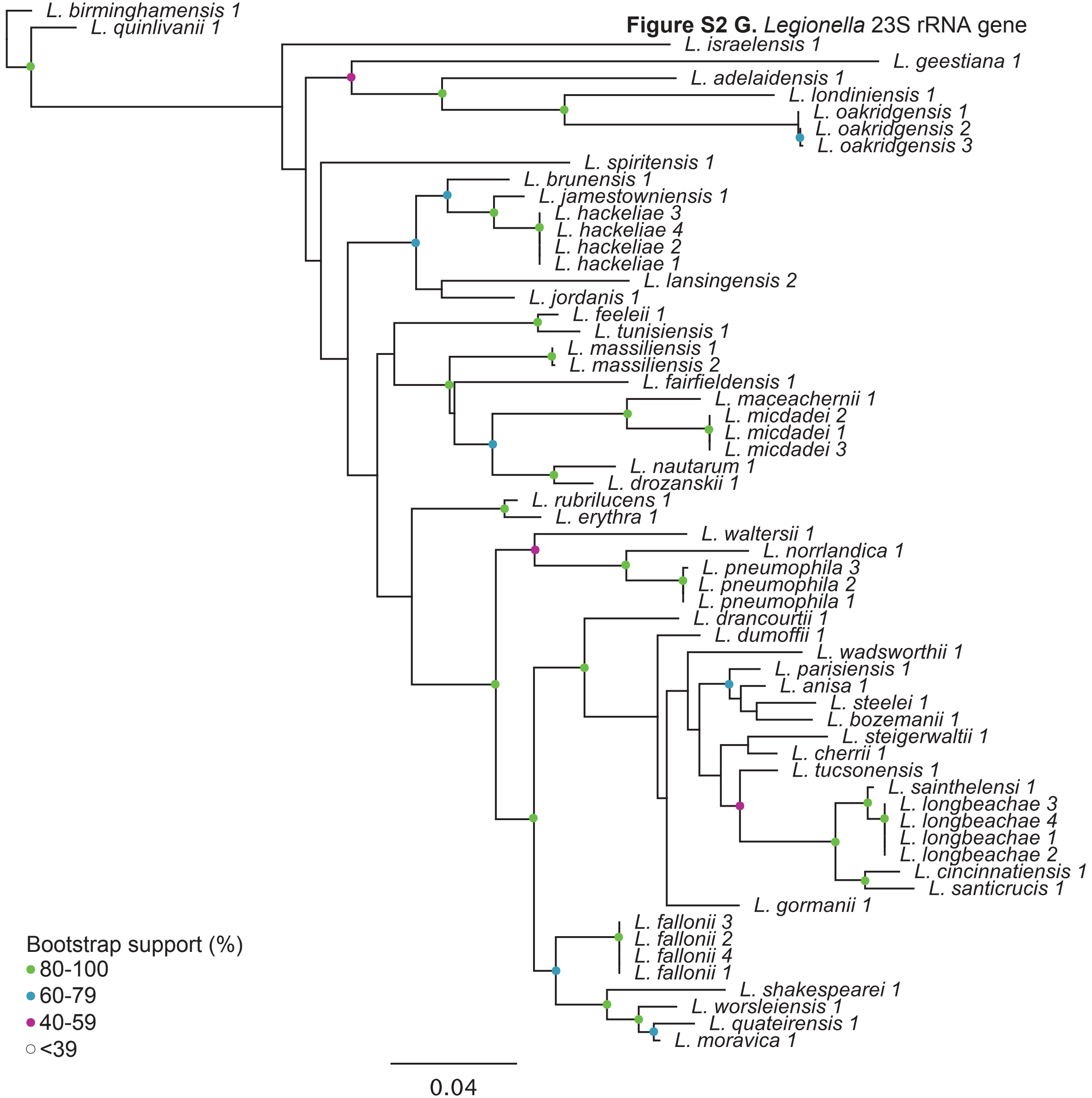

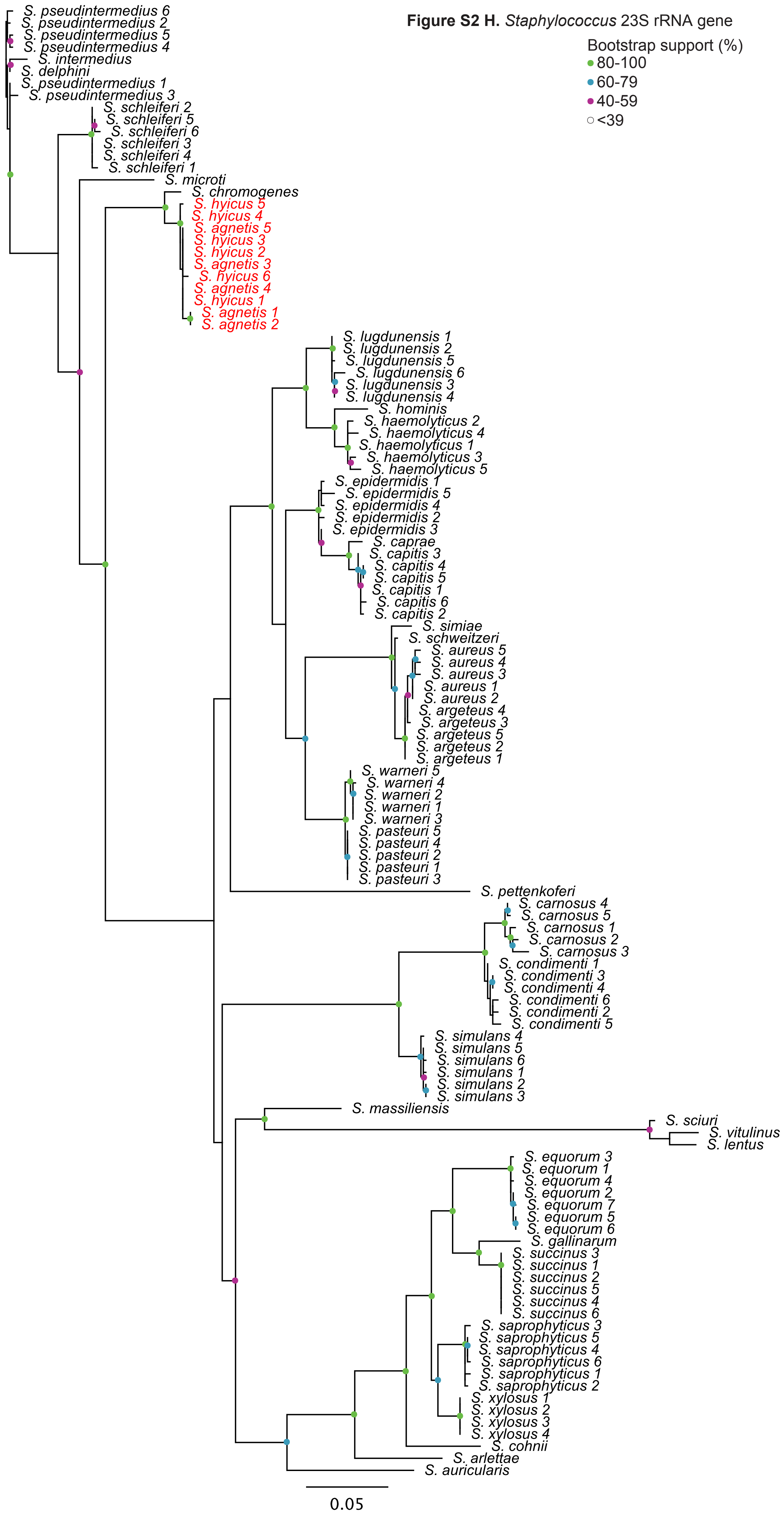

Figure S2 H. *Staphylococcus* 23S rRNA gene

Figure S2 I. *Campylobacter* 5S rRNA gene

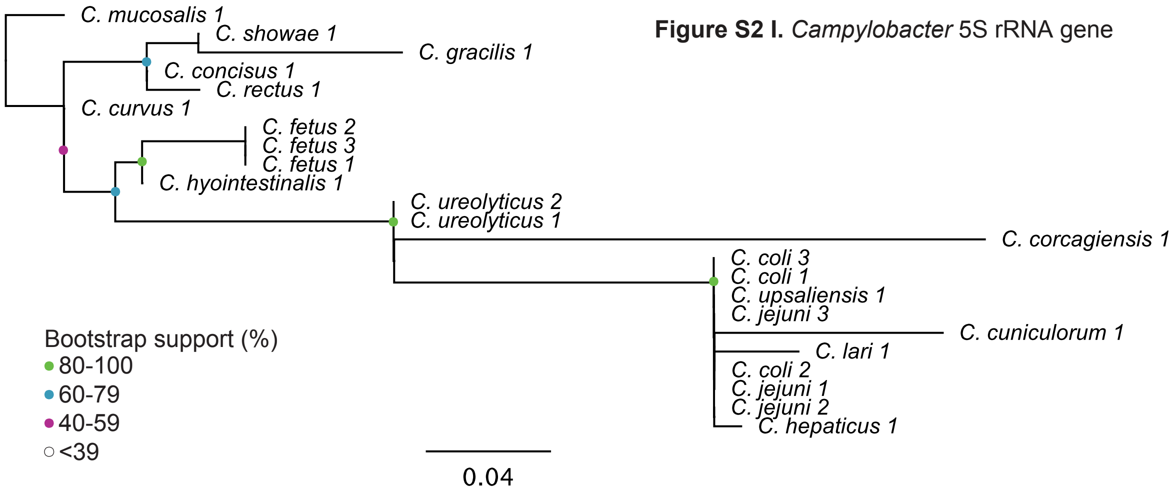

Figure S2 J. *Clostridium* 5S rRNA gene

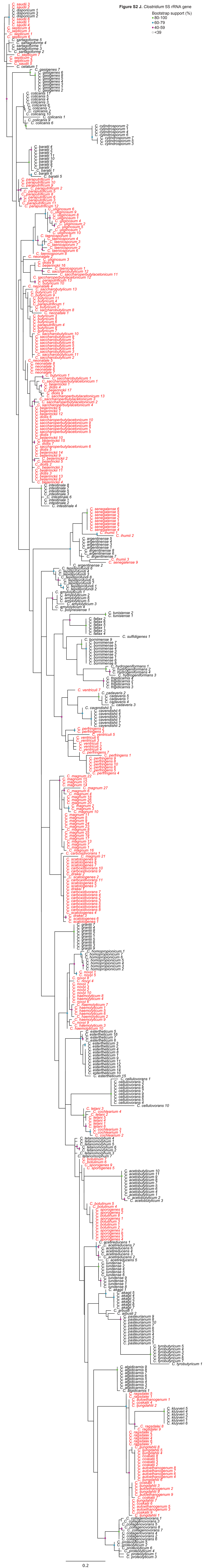

Figure S2 K. *Legionella* 5S rRNA gene

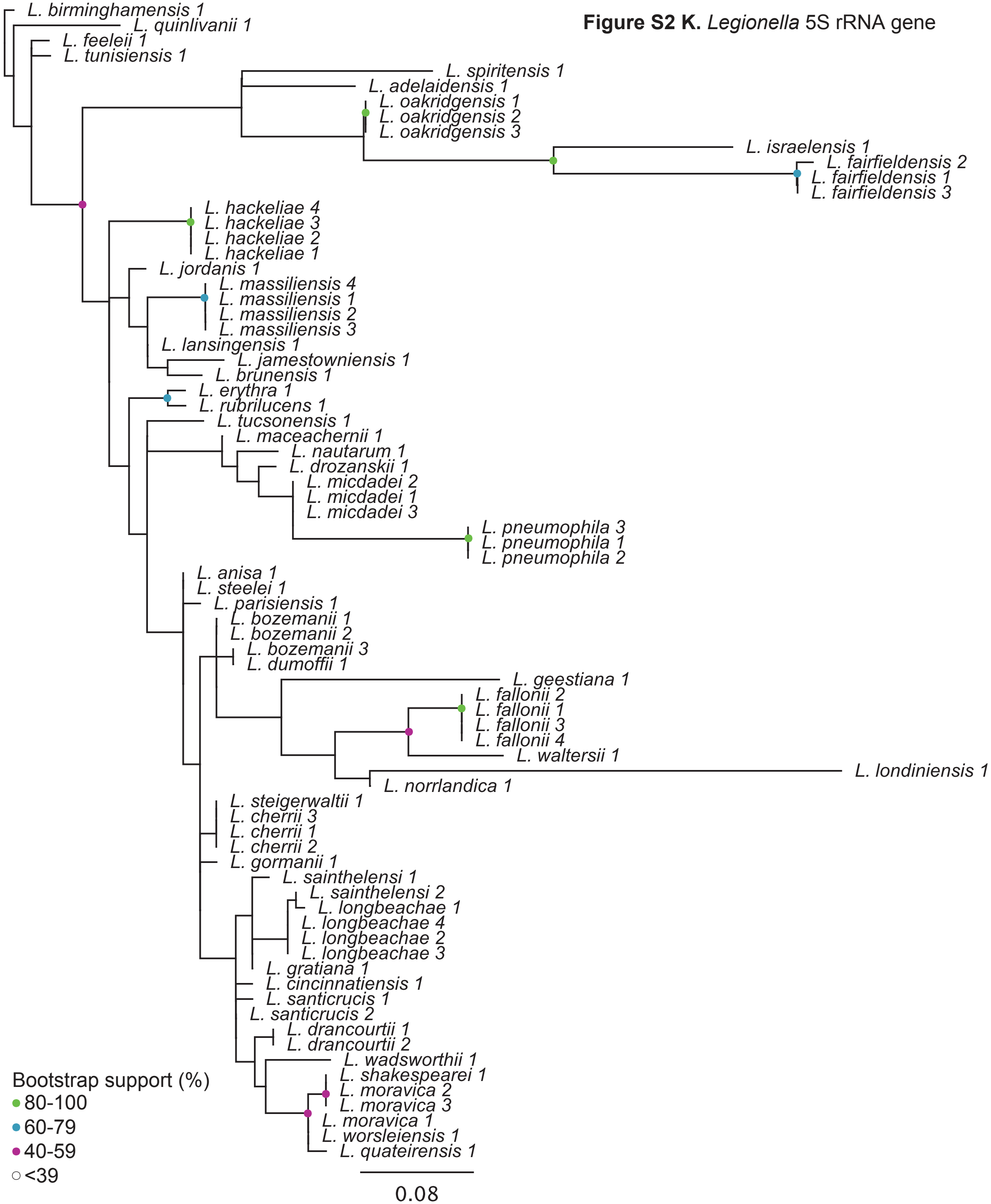

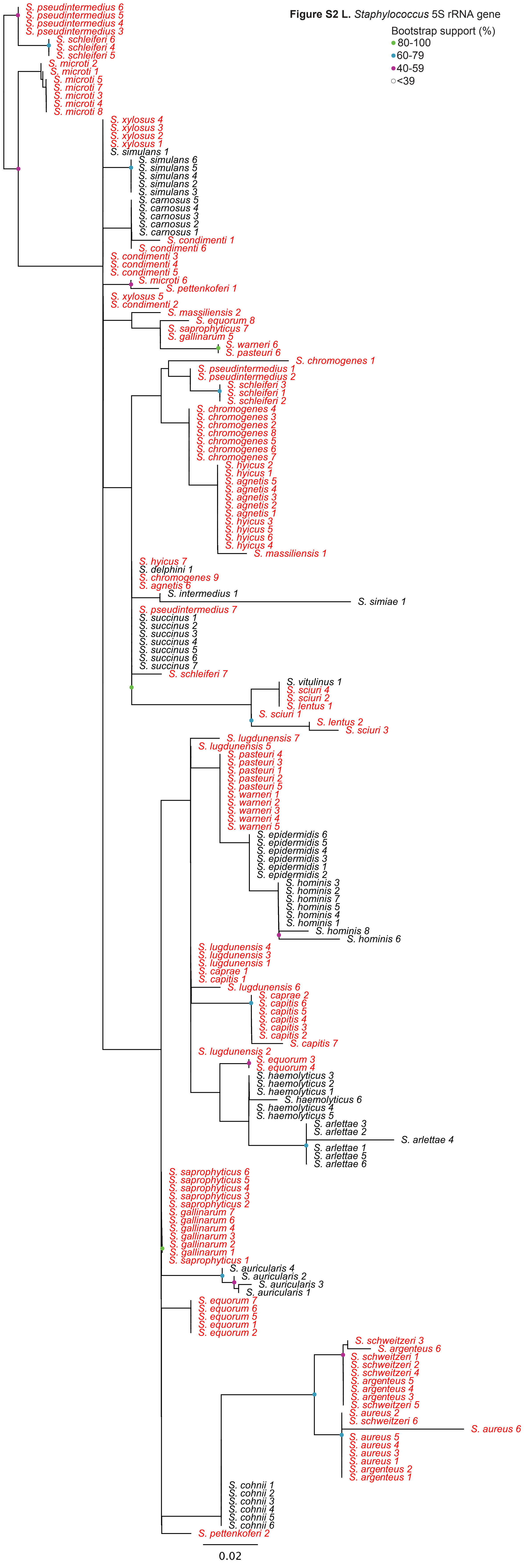

Figure S2. 16S rRNA gene ML phylogenies including all copies of the gene for A. *Campylobacter*, B. *Clostridium*, C. *Legionella*, D. *Staphylococcus*. 23S rRNA gene ML phylogeny including all copies of the gene for E. *Campylobacter*, F. *Clostridium*, G. *Legionella*, H. *Staphylococcus*. 5S rRNA gene ML phylogeny including all copies of the gene for I. *Campylobacter*, J. *Clostridium*, K. *Legionella*, L. *Staphylococcus*. Putative events of HGT are denoted by red labeled taxa. Numbers next to taxa names indicate copy number. Bootstrap values are based on 200 bootstrap replicates.

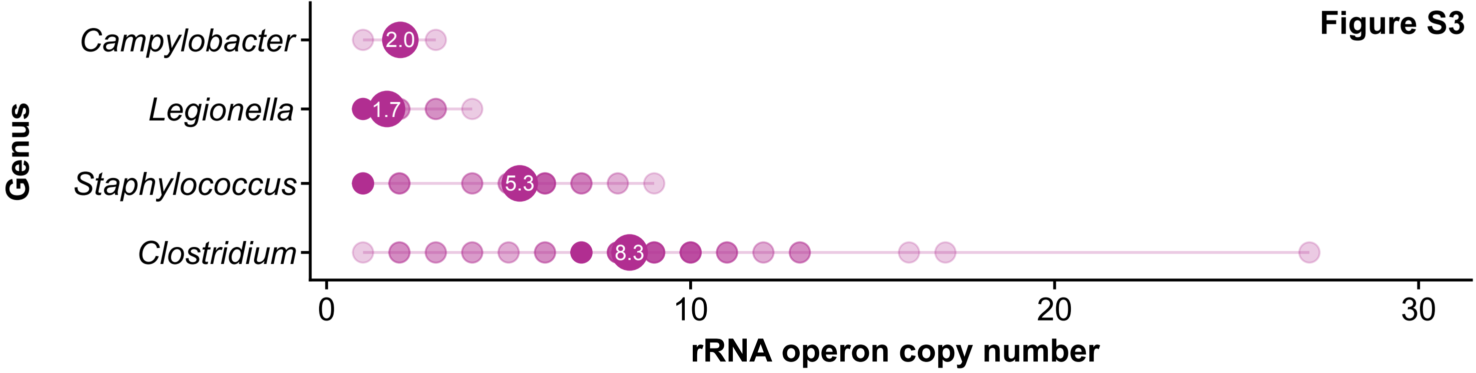

Figure S3. rRNA operon copy numbers for species within each genus. Dot shading represents the number of species for an associated operon copy number. Within each genus, the level of shading is relative to the maximum and minimum number of species possessing any operon copy number. Larger dots indicate the average number of operon copies.

Figure S4

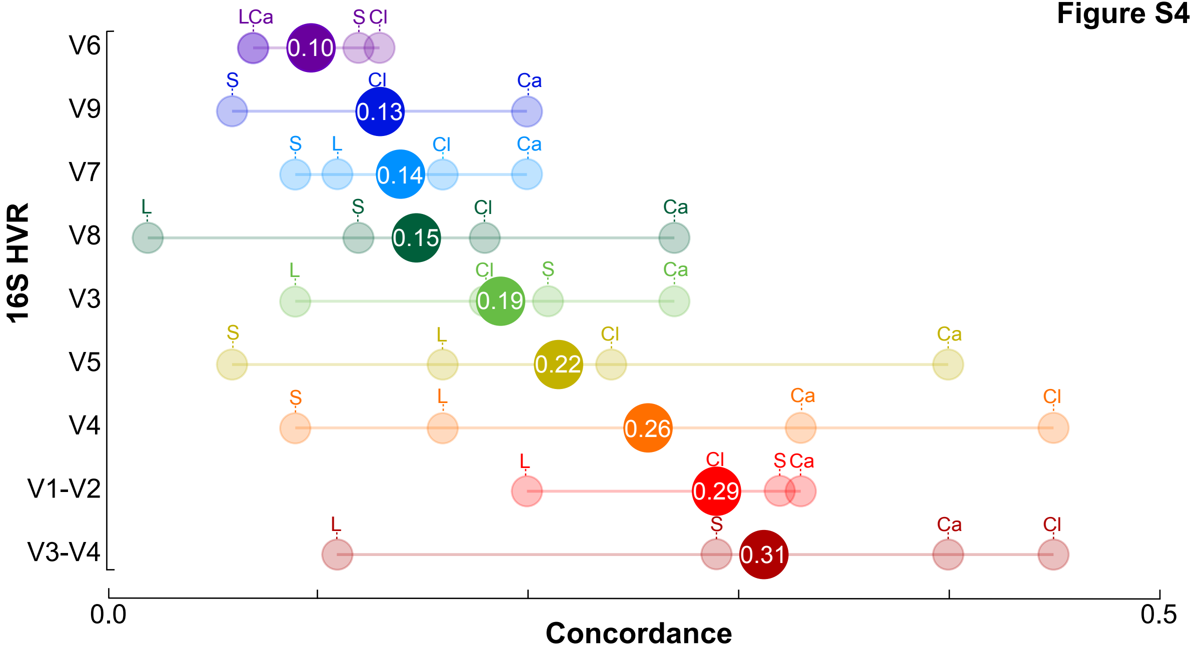

Figure S4. Dot plot showing levels concordance with the species phylogeny for each 16S rRNA gene hypervariable region (16S HVR) for each genus. Genus labels follow Figure 2. Average concordance for each region is shown by larger dots.

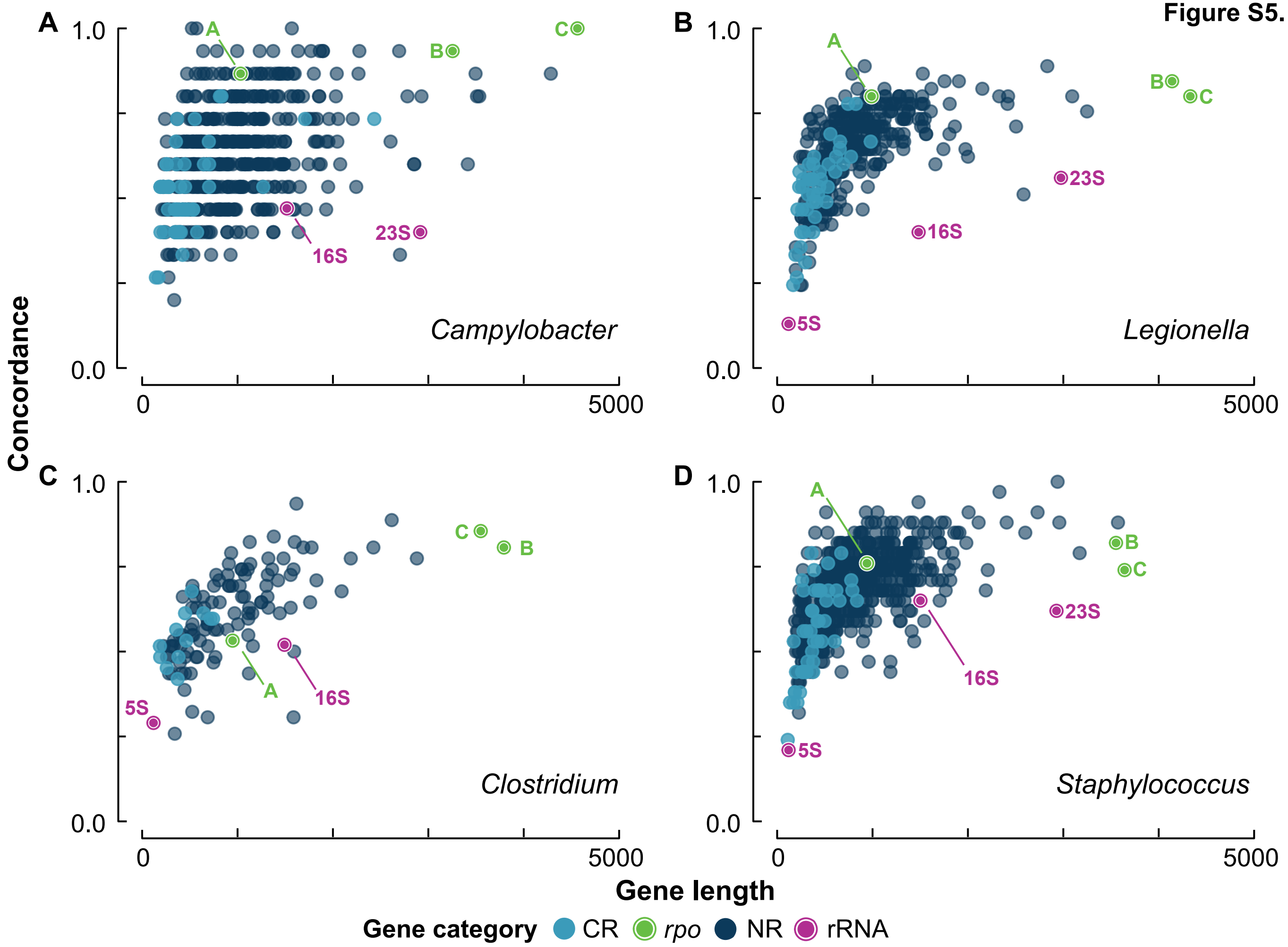

Figure S5. Dot plot showing the relationship between concordance with the species phylogeny and core gene length. Gene and gene category labels and coloring follow Figure 1.

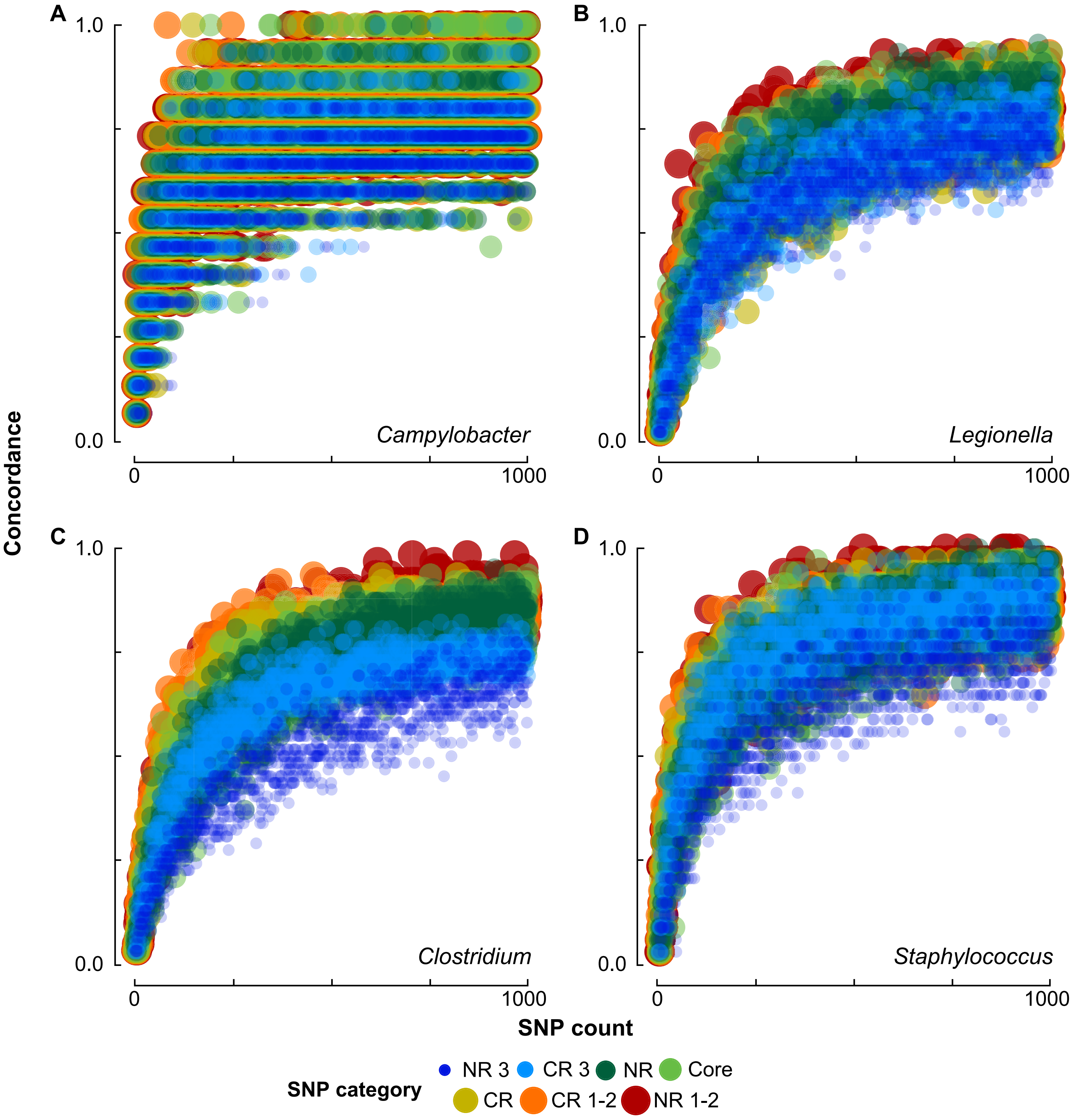

Figure S6. Dot plot showing the relationship between core gene concordance with the species phylogeny and SNP count. For each core gene alignment, delineated into SNP categories, SNPs were randomly extracted in an iterative process to build 1,000 alignments ranging in size from 1 to 1,000 nt. Concordance for each alignment was plotted against its SNP count. Different dot sizes were used for clarity.

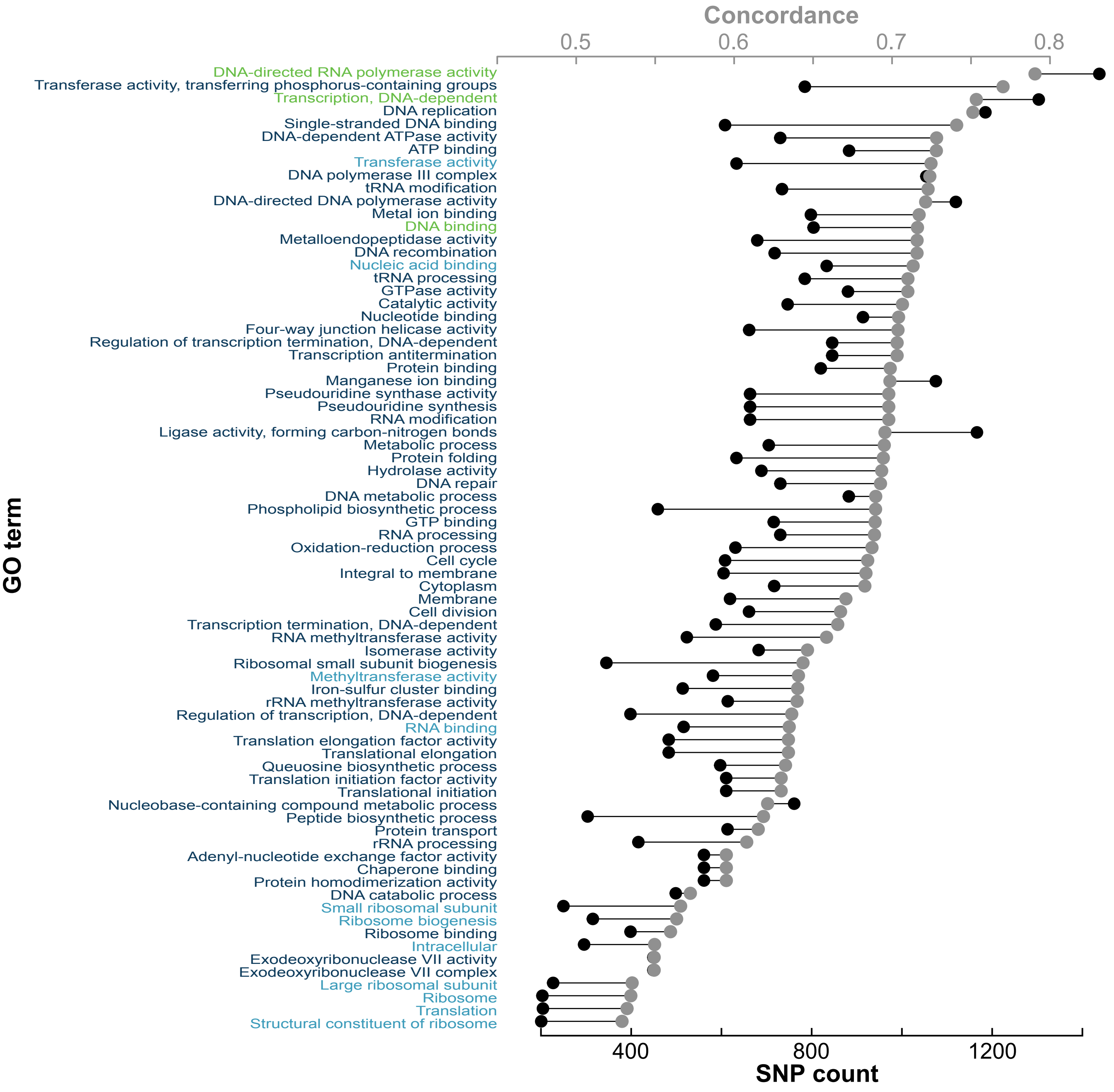

Figure S7. Dot plot showing average concordance (grey) and SNP count (black) for genes assigned universal biochemical GO terms. Text coloring indicats GO terms assigned to *rpo* genes (green), coding ribosomal genes (light blue), and non-ribosomal genes (dark blue).

Figure S8 A. Inter-genus species phylogeny

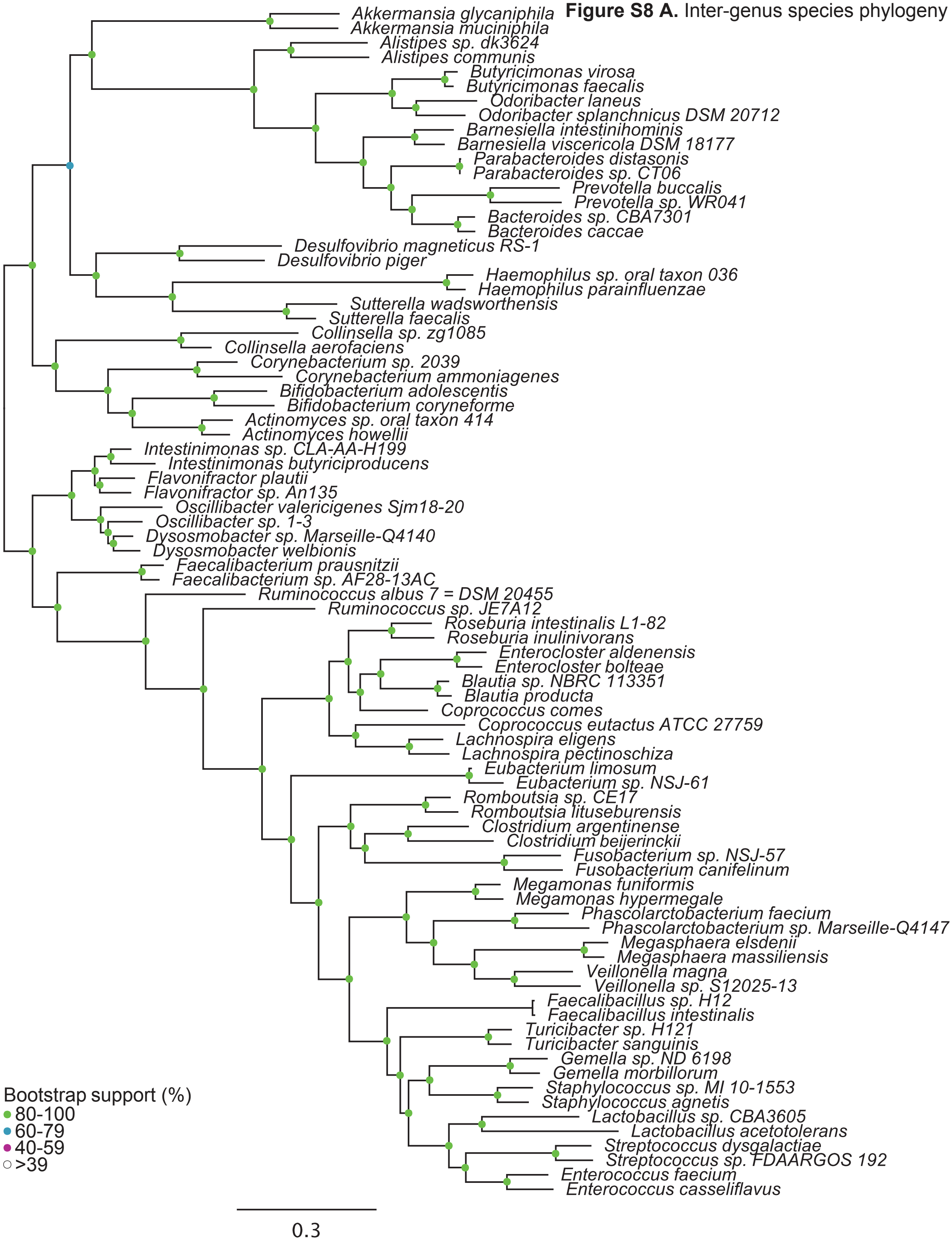

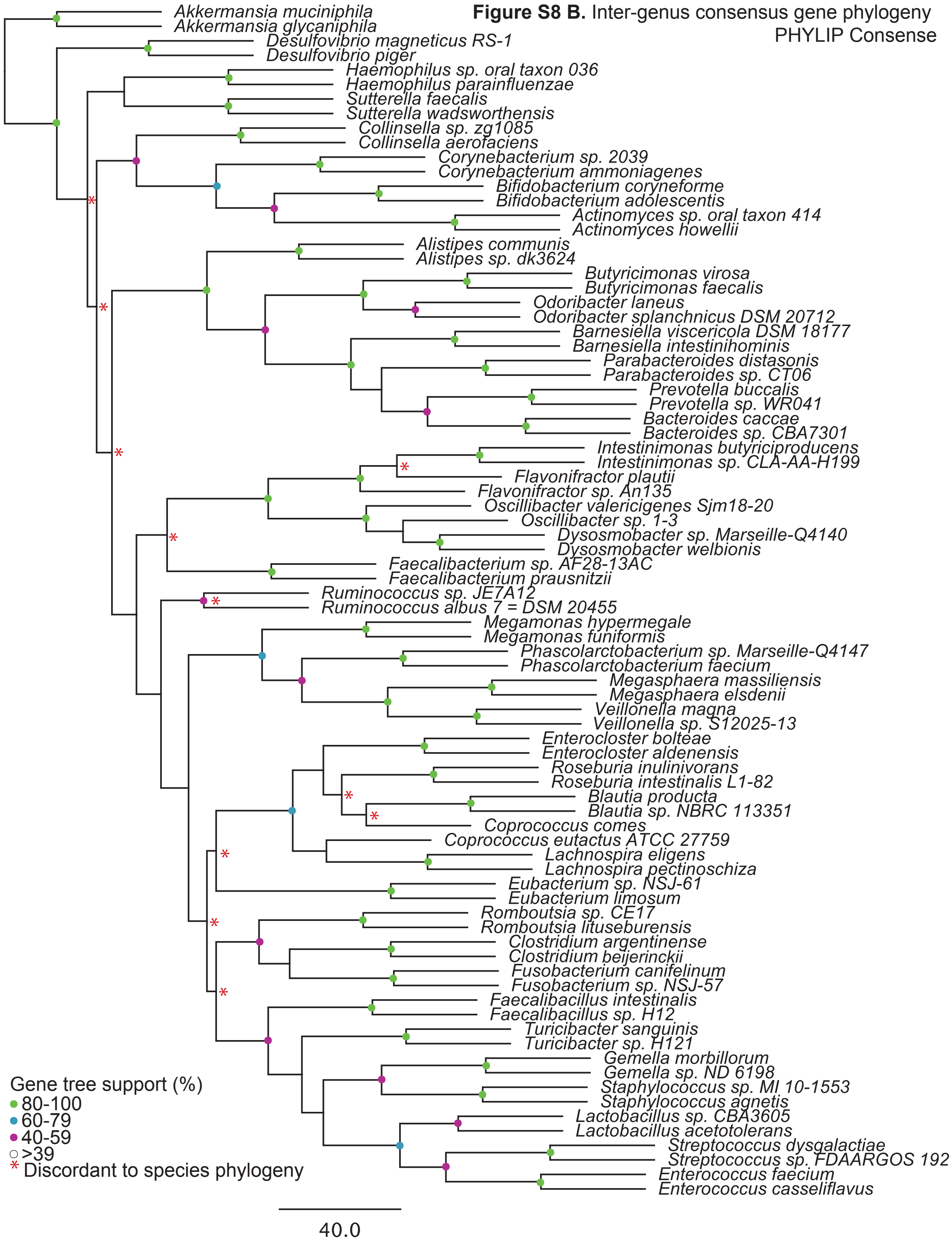

Figure S8. Concatenated maximum-likelihood and consensus species phylogenies for inter-genus level analysis.

Figure S9

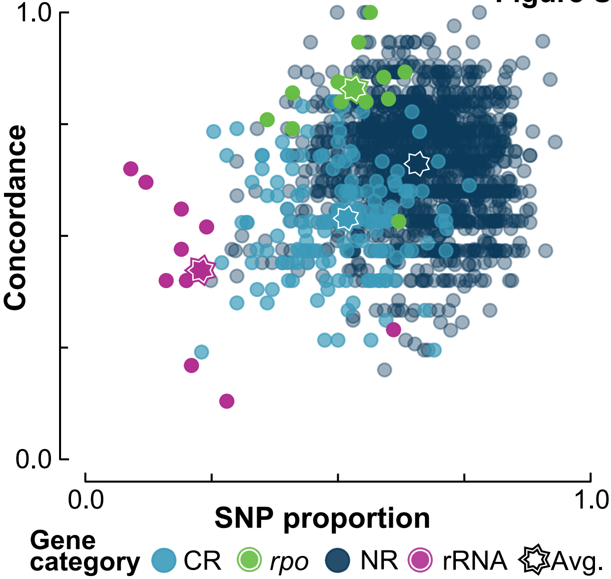

Figure S9. Dot plot showing the relationship between concordance with the species phylogeny and core gene SNP proportion. All genera are combined and the average SNP proportions for each gene category are shown with a star. Gene and gene category labels and coloring follow Figure 1.
